# Global gene expression profiling of brown to white adipose tissue transformation in sheep reveals novel transcriptional components linked to adipose remodeling

**DOI:** 10.1186/s12864-015-1405-8

**Published:** 2015-03-19

**Authors:** Astrid L Basse, Karen Dixen, Rachita Yadav, Malin P Tygesen, Klaus Qvortrup, Karsten Kristiansen, Bjørn Quistorff, Ramneek Gupta, Jun Wang, Jacob B Hansen

**Affiliations:** Department of Biology, University of Copenhagen, DK-2100 Copenhagen, Denmark; Department of Biomedical Sciences, University of Copenhagen, DK-2200 Copenhagen, Denmark; Department of Systems Biology, Center for Biological Sequence Analysis, Technical University of Denmark, DK-2800 Kongens Lyngby, Denmark; Department of Veterinary Clinical and Animal Sciences, University of Copenhagen, DK-1870 Frederiksberg, Denmark; BGI-Shenzhen, Shenzhen, 518083 China; Princess Al Jawhara Center of Excellence in the Research of Hereditary Disorders, King Abdulaziz University, Jeddah, 21589 Saudi Arabia; Macau University of Science and Technology, Avenida Wai Long, Taipa, Macau, 999078 China

**Keywords:** BAT, Brite/beige adipose tissue, Global gene expression profiling, Mitochondrial number, Sheep, Transcription factors, UCP1, WAT

## Abstract

**Background:**

Large mammals are capable of thermoregulation shortly after birth due to the presence of brown adipose tissue (BAT). The majority of BAT disappears after birth and is replaced by white adipose tissue (WAT).

**Results:**

We analyzed the postnatal transformation of adipose in sheep with a time course study of the perirenal adipose depot. We observed changes in tissue morphology, gene expression and metabolism within the first two weeks of postnatal life consistent with the expected transition from BAT to WAT. The transformation was characterized by massively decreased mitochondrial abundance and down-regulation of gene expression related to mitochondrial function and oxidative phosphorylation. Global gene expression profiling demonstrated that the time points grouped into three phases: a brown adipose phase, a transition phase and a white adipose phase. Between the brown adipose and the transition phase 170 genes were differentially expressed, and 717 genes were differentially expressed between the transition and the white adipose phase. Thirty-eight genes were shared among the two sets of differentially expressed genes. We identified a number of regulated transcription factors, including NR1H3, MYC, KLF4, ESR1, RELA and BCL6, which were linked to the overall changes in gene expression during the adipose tissue remodeling. Finally, the perirenal adipose tissue expressed both brown and brite/beige adipocyte marker genes at birth, the expression of which changed substantially over time.

**Conclusions:**

Using global gene expression profiling of the postnatal BAT to WAT transformation in sheep, we provide novel insight into adipose tissue plasticity in a large mammal, including identification of novel transcriptional components linked to adipose tissue remodeling. Moreover, our data set provides a useful resource for further studies in adipose tissue plasticity.

**Electronic supplementary material:**

The online version of this article (doi:10.1186/s12864-015-1405-8) contains supplementary material, which is available to authorized users.

## Background

Two types of adipose tissue exist based on morphological appearance and biological function. White adipose tissue (WAT) stores energy in the form of triacylglycerol (TAG) for later release and use by other tissues, whereas brown adipose tissue (BAT) metabolizes fatty acids and glucose for heat production. Thermogenesis through uncoupled respiration in BAT depends on a high mitochondrial density and expression of uncoupling protein 1 (UCP1) [1]. Larger mammals such as primates and ruminants are born fully developed and able to thermoregulate minutes after birth due to the presence of relatively large amounts of functional BAT, which gets activated at birth. The majority of this innate BAT disappears after birth and is replaced by WAT [1-3]. Besides brown adipocytes in BAT, another type of thermogenic adipocytes exists: the brite (brown-in-white) or beige adipocytes, which are known to be recruited in subcutaneous WAT of rodents in response to cold exposure or stimulation with β-adrenergic agonists [4,5]. The developmental origin of brite/beige adipocytes and their contribution to human BAT are still a matter of debate [6,7].

Rodents as opposed to larger mammals are born with immature BAT which matures only postnatally and is largely retained throughout life [1,8]. It is relevant to understand the brown to white adipose tissue remodeling in large mammals, as it is likely to mimic the transition occurring in human infants. The most frequently studied adipose tissue transition in a large mammal is the postnatal transformation of the perirenal adipose tissue in sheep. Around the time of birth, all visceral adipose depots in lambs are brown of nature [3,9]. Lambs are normally born with approximately 30 g perirenal adipose tissue constituting 80% of all their adipose tissue. The brown characteristics of the perirenal adipose depot change dramatically to a white adipose phenotype within a few weeks after birth [3,9]. Although some gene expression details have been reported [10,11], relatively little is known about this transition at the molecular level.

Here we report a comprehensive time course analysis of the postnatal BAT to WAT transformation process of the perirenal adipose depot in lambs, including histological, biochemical and molecular examination as well as analyses of global gene expression profiles. We provide evidence for dramatic changes in mitochondrial function and fatty acid metabolism during the adipose remodeling and we identified a number of transcriptional components linked to this adipose tissue transformation process.

## Methods

### Animals and tissues

Experimental procedures were in compliance with guidelines laid down by the Danish Inspectorate of Animal Experimentation. Lambs from cross-bred ewes (Texel x Gotland) in their second or third parturition sired by purebred Texel ram, born and raised at a commercial farm in Denmark, were used. During gestation ewes were fed hay ad libitum, 200 g barley and 200 g commercial concentrate per day. Ewes were housed in groups of 40 until lambing. After lambing they were housed individually for 2 days and subsequently housed in groups of 20 until they were transferred to pasture approximately one week after lambing. The ewe-reared lambs were kept on pasture being a mixture of 70% ray grass and 30% white clover. Lambs were killed by bolt pistol and bled by licensed staff. Perirenal adipose tissue was carefully dissected and frozen in liquid nitrogen for biochemical or molecular analyses or fixed for histology as described below. Lambs at the following ages (day relative to the time of birth) were used: −2 (n = 4), 0 (n = 5), 0.5 (n = 5), 1 (n = 5), 2 (n = 5), 4 (n = 5), 14 (n = 5), 30 (n = 5) and 60 (n = 5). Live weights of the lambs were kept similar within groups. The ratio of females to males was similar (0.3) in the three phases of the transformation.

### Hematoxylin-eosin (HE) staining

Samples were fixed in 4% neutral buffered formaldehyde (pH 7.4) at room temperature for 24 h and subsequently at 4°C until preparation. The tissue was processed to paraffin and sectioned in 4 μm sections. HE staining was performed according to standard procedures.

### Transmission electron microscopy (TEM)

Samples were fixed in Karnowsky’s fixative (2% paraformaldehyde and 2.5% glutaraldehyde in 0.08 M cacodylate buffer, pH 7.4) for 3–5 days at room temperature and subsequently stored in 0.08 M cacodylate buffer at 4°C until further processing. The samples were rinsed three times in 0.15 M Sorensen’s phosphate buffer (pH 7.4) and subsequently post fixed in 1% OsO_4_ in 0.12 M sodium cacodylate buffer (pH 7.4) for 2 h. The specimens were dehydrated in graded series of ethanol, transferred to propylene oxide and embedded in Epon according to standard procedures. Ultra-thin sections were cut with a Reichert-Jung Ultracut E microtome and collected on single slot copper grids with Formvar supporting membranes. Sections were stained with uranyl acetate and lead citrate and examined with a Philips CM-100 transmission electron microscope, operated at an accelerating voltage of 80 kV. Digital images were recorded with a SIS MegaView2 camera and the analySIS software package.

### Reverse transcription-quantitative polymerase chain reaction (RT-qPCR)

Tissues were homogenized in TRIzol (Life Technologies) using a Dispomix (Xiril) and total RNA was purified. Reverse transcriptions were performed in 25 μl reactions containing 1st Strand Buffer (Life Technologies), 2 μg random hexamers (Bioline), 0.9 mM of each dNTP (Sigma-Aldrich), 20 units of RNaseOUT (Life Technologies), 1 μg of total RQ1 DNase (Promega)-treated RNA and 200 units of Moloney murine leukemia virus reverse transcriptase (Life Technologies). Reactions were left for 10 min at room temperature, followed by incubation at 37°C for 1 h. After cDNA synthesis, reactions were diluted with 50 μl of water and frozen at −80°C. The cDNA was analyzed by RT-qPCR using the Stratagene Mx3000P QPCR System. Each PCR mixture contained, in a final volume of 20 μl, 1.5 μl of 1st strand cDNA, 10 μl of SensiFAST™ SYBR Lo-ROX Kit (Bioline) and 2 pmol of each primer (Additional file 1: Table S1). All reactions were run using the following cycling conditions: 95°C for 10 min, then 40 cycles of 95°C for 15 s, 55°C for 30 s and 72°C for 15 s. PCR was carried out in 96-well plates and each sample was run in duplicate. Target gene mRNA expression was normalized to the stably expressed β-actin (*ACTB*) mRNA.

### Protein extracts and immunoblotting

Tissues were homogenized in a GG-buffer (pH 7.5) containing 25 mM glycyl-glycin, 150 mM KCl, 5 mM MgSO_4_ and 5 mM ethylenediaminetetraacetic acid (EDTA) as well as freshly added dithiothreitol (1 mM), bovine serum albumin (0.02%) and Triton X-100 (0.1%). Homogenization was performed with a TissueLyser (QIAGEN) using 5 mm stainless steel beads, and homogenates were subsequently frozen in liquid nitrogen. Protein concentrations were determined by the Lowry method [12] and equal amounts of protein from each animal were pooled according to age and diluted in a buffer containing 2.5% SDS and 10% glycerol. Proteins were separated on 4-12% Bis-Tris gradient gels (NuPAGE, Life Technologies), blotted onto Immobilon PVDF membranes (Millipore) and stained with Amido Black 10B (Sigma-Aldrich). Membranes were blocked in Tris-buffered saline (pH 7.4) or phosphate-buffered saline (pH 9.0) with 5% nonfat dry milk and 0.1% Tween 20 (Sigma-Aldrich) and subsequently probed with antibodies. Primary antibodies used were against transcription factor IIB (TFIIB) (sc-225) (Santa Cruz Biotechnology), ATP synthase β (ATP5B) (ab14730) (Abcam) and UCP1 (ab10983) (Abcam). Secondary antibodies were horseradish peroxidase-conjugated (Dako). Enhanced chemiluminescence (Biological Industries) was used for detection.

### Quantification of relative mitochondrial DNA (mtDNA) copy numbers

Relative mtDNA amount (copy number) was measured as the ratio between mtDNA and nuclear DNA (nDNA). Tissues were homogenized using a TissueLyser (QIAGEN) in lysis buffer containing 100 mM Tris-base (pH 8.0), 5 mM EDTA (pH 8.0), 0.2% sodium dodecyl sulphate, 200 mM NaCl and 100 mg/ml proteinase K and incubated overnight at 55°C with rotation. DNA was precipitated with two volumes of 99% ethanol and fished out with inoculation loops, washed in 70% ethanol and dissolved in Tris-EDTA buffer containing 10 mg/ml RNase A at 55°C overnight. DNA concentrations were determined on the Eppendorf BioPhotometer at 260 nm and 50 ng DNA was used for qPCR. PCR reactions and cycling conditions were as described above, and primers were against cytochrome c oxidase I (*MT-CO1*) (mtDNA) and suppression of tumorigenicity 7 (*ST7*) (nDNA) (Additional file 1: Table S1).

### Citrate synthase (CS) activity

Tissue homogenates (10%) were generated in GG-buffer (pH 7.5) as described above. Homogenates were thawed on ice and centrifuged at 4°C at 20,000 g for 2 min. Supernatants were used for activity measurements. CS activity was measured spectrophotometrically at 25°C and 412 nm in CS buffer containing 100 mM Tris-base (pH 8.0), 10 mM 5,5′-dithiobis(2-nitrobenzoic acid), 5 mM acetyl-CoA and 50 mM oxaloacetic acid and activity was measured as described [13]. Each sample was measured in duplicate and the mean was used for subsequent calculations. Activities were normalized to the amount of total protein determined by the Lowry method [12].

### Statistical analyses of qPCR data

The time course study was analyzed for statistical significance using one-way ANOVA and Student’s t-test with Bonferroni correction for multiple testing as post hoc test. A *p*-value < 0.05 was considered statistically significant.

### Targeted RNA-sequencing and data analysis

#### Isolation of mRNA and synthesis of first strand cDNA

Equal amounts of total RNA from perirenal adipose tissue from lambs at the same age (days −2, 0, 0.5, 1, 2, 4, 14) were pooled. mRNA was isolated from 4 μg of total RNA by magnetic oligo(dT) beads, which was used to synthesize bead-bound cDNA, according to the instructions of the manufacturer (Illumina).

#### Tag library construction

The library for digital gene expression analysis was constructed according to the instructions of the manufacturer (Illumina). Bead-bound cDNA was digested with NlaIII, followed by ligation of the GEX adapter 1 to the bead-bound NlaIII-digested cDNA. This was then digested with MmeI, releasing the GEX adaptor 1 linked to 17 bp cDNA from the beads. The released fragment was ligated to GEX adapter 2. The 17 bp tags of cDNA were PCR amplified using two primers that anneal to the two adapters. The resultant tag library was used for Illumina sequencing.

#### Data analysis for RNA-seq data

Quality control, trimming and adapter removal was performed using FastQC [14] and fastx_clipper from the FASTX-Toolkit [15]. The 4 bases CATG were added to the 5’ end of reads to increase the specificity of mapping. BWA [16] was employed for the alignment and mapping of reads to the sheep genome. The October 2012 release of the sheep genome v3 [17] was used as reference for the alignment. Mapped reads were sorted and indexed with samtools [18]. Since the RNA-seq data was used for expression quantification, duplicated reads were not removed. HT-Seq [19] was used for counting mapped reads per annotated gene, using the annotation file provided for the sheep genome v3 release. DESeq [20] and R [21] were used for the post processing and statistical analysis of these read counts.

#### Principal component analysis (PCA) and hierarchical clustering of time points

A two-dimensional PCA plot was employed to visualize the overall effect of experimental covariates. Hierarchical clustering of the total gene expression was performed using a distance matrix to assess the relationship between the samples and identify clusters amongst the time points.

#### Grouping of time points

Based on the PCA and hierarchical clustering of the total gene expression, days −2 and 0 were used as replicates of the “brown adipose state”, days 0.5, 1, 2 and 4 as replicates of the “transition state” and day 14 as the “white adipose state”. Using the DESeq package of Bioconductor [22], differentially expressed genes were found between the brown adipose and the transition state as well as between the transition and the white adipose state. For the differential gene expression between “transition state” and “white adipose state”, the partial replicate function of DESeq for calculation of dispersion of the genes was used. This method ignores the sample from day 14 when estimating dispersion for the genes and would use only days 0.5, 1, 2, 4 for calculating the dispersion per gene, which is an indicator of variability in that gene, and is used to fit the negative binomial model on the count data. Heatmaps representing clustering for the differentially expressed genes were created using the ggplots [23] package in R. The sheep proteins were queried against the human non-redundant protein database using BLAST [24] to find human homologous genes for further functional analysis. Reciprocal BLAST, a computation method used to countercheck the BLAST results, was employed to filter the correct mapping between the sheep and human proteins. The BioMart tool on Ensemble version 72 [25] was used for gene identification and conversion, and for obtaining Human Genome Organization (HUGO) Gene Nomenclature Committee (HGNC) approved gene names for the human homologous proteins. UniProt [26] was used to annotate the proteins for function, transcriptional activity and subcellular localization. GO term enrichment was done using ExPlain™ [27] from BIOBASE Corporation. The Explain tool uses false discovery rate (FDR) correction for finding significantly enriched GO classes. For the two sets of differentially expressed genes obtained from DESeq analysis, Enrichr [28] was used to find enrichment of transcription factors and the corresponding targets for the differentially expressed transcription factors from the Transfac [29] and Jasper [30] databases. Enrichr applies a Fisher’s exact test to calculate significance, which is further corrected for multiple testing and the resulting adjusted *p-*values are used for selecting significant transcription factors for the differentially expressed genes.

## Results

### Characterization of the postnatal brown to white adipose transformation

**Figure 1:**
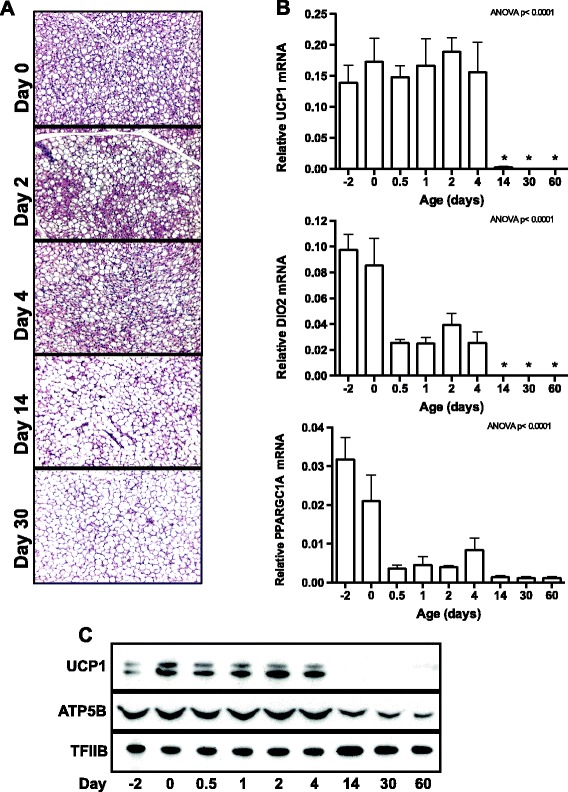
**Characterization of the postnatal brown to white adipose transformation. (A)** Hematoxylin-eosin (HE) staining of perirenal adipose tissue at postnatal days 0, 2, 4, 14 and 30. Representative HE-stained sections are shown for the indicated time points (n = 5). **(B)** Total RNA was isolated from perirenal adipose tissue and used for RT-qPCR analysis. Relative expression was measured for uncoupling protein 1 (*UCP1*), type II iodothyronine deiodinase (*DIO2*) and peroxisome proliferator-activated receptor γ (PPARG) co-activator 1α (*PPARGC1A*). The mRNA expression levels were normalized to expression of β-actin (*ACTB*). Data are mean + SEM (n =4-5); *, *p* < 0.05 vs. day 0. **(C)** The level of uncoupling protein 1 (UCP1) and ATP synthase subunit β (ATP5B) was determined by immunoblotting on protein pools, one for each day during the time course. Transcription factor IIB (TFIIB) was used as a loading control.

At birth (designated day 0) the perirenal adipose tissue macroscopically appeared dark brown. The brown color fainted steadily during the time course, and the tissue ended up being white in appearance at postnatal days 30 and 60 (data not shown). Accompanying the “whitening”, the volume of the tissue gradually increased (data not shown). HE-stained sections were prepared from all lambs, and representative sections from days 0, 2, 4, 14 and 30 are presented in Figure 1A. In the first week of life, the tissue was an apparent mixture of brown adipocytes with multilocular lipid droplets and white adipocytes with large unilocular lipid droplets. The perirenal adipose tissue contained by appearance mostly brown adipocytes at early ages (days 0 to 4), whereas white adipocytes were predominant from day 14.

To approach the BAT to WAT transformation in molecular terms, we measured mRNA and protein levels of selected marker genes by RT-qPCR and immunoblotting, respectively (Figure 1B and C). Expression of *UCP1*, the brown adipocyte-specific key thermogenic factor, was high and relatively stable until day 4, after which it became nearly undetectable. The BAT-enriched factors type II iodothyronine deiodinase (*DIO2*) and peroxisome proliferator-activated receptor γ (PPARG) co-activator 1α (*PPARGC1A*) were also highly expressed at days −2 and 0, but displayed a faster and stepwise decrease in expression, being considerably reduced already at days 0.5 and 1 and poorly expressed after day 4 (Figure 1B).

In summary, at the level of macroscopic, microscopic and molecular analyses, we observed the expected postnatal transformation of BAT to WAT.

### Mitochondrial density declined during brown to white adipose transformation

**Figure 2:**
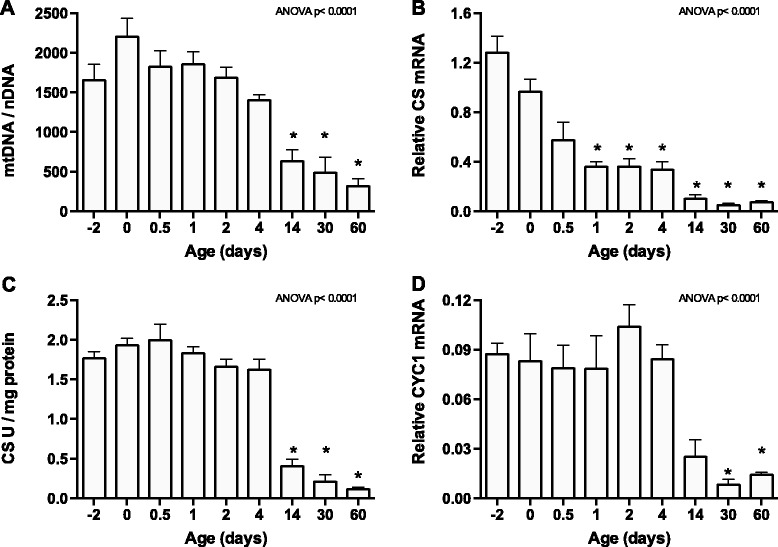
**Mitochondrial density declines during brown to white adipose transformation. (A)** Total DNA was isolated from perirenal adipose tissues and analyzed by qPCR with primers specific for mtDNA (cytochrome c oxidase I (*MT-CO1*)) and nDNA (suppression of tumorigenicity 7 (*ST7*)). The relative mtDNA copy number was obtained as the ratio of *MT-CO1* to *ST7* levels. **(B)** Total RNA was isolated from perirenal adipose tissue and used for RT-qPCR analysis. Relative expression was measured for citrate synthase (*CS*). The mRNA expression levels were normalized to expression of β-actin (*ACTB*). **(C)** Enzyme activity (U) of CS was determined spectrophotometrically in homogenates of perirenal adipose tissue and normalized to protein content. **(D)** Relative expression of cytochrome c1 (*CYC1*) was measured by RT-qPCR as described in panel B. Data are mean + SEM (n = 4–5); *, *p* < 0.05 vs. day 0.

The ultra-structure of the perirenal adipose tissue was investigated at selected days by TEM (Additional file 2: Figure S1A). TEM confirmed the mixed presence of multilocular and unilocular adipocytes at days 0 to 4 and the predominant presence of the latter at day 14. Adipocyte mitochondrial density was very high at days 0 to 4 and appeared lower at day 14. To estimate mitochondrial density quantitatively, we determined mtDNA content by qPCR as the ratio of mtDNA and nDNA (Figure 2A). This ratio decreased approximately 7-fold between days 0 and 60, indicating that the number of mitochondria per cell diminished during the BAT to WAT transformation.

The ultra-structural observations and the mtDNA/nDNA ratios prompted us to investigate more carefully gene expression of relevance for mitochondrial abundance and function. The mRNA levels of the tricarboxylic acid (TCA) cycle enzyme *CS* decreased gradually during the time course (Figure 2B). CS activity, on the other hand, was high and stable until day 4, after which it dropped (Figure 2C). Two other mitochondrial genes were analyzed: the electron transporter cytochrome c1 (*CYC1*) and *ATP5B*. Levels of *CYC1* mRNA (Figure 2D) and ATP5B protein (Figure 1C) displayed a time profile similar to that of CS activity.

A number of nuclear transcription factors and co-regulators regulate expression of genes encoding mitochondrial proteins. These factors include PGC-1 family members, nuclear respiratory factor 1 (NRF1) and a number of nuclear receptors. In addition to *PPARGC1A* (see Figure 1B), we measured the expression of *PPARGC1B*, *PPARA* (also known as *NR1C1*), estrogen-related receptor α (*ERRA*, also known as *NR3B1*) and *NRF1* by RT-qPCR (Additional file 2: Figure S1B). The expression pattern of *PPARGC1B* and *ERRA* was similar to *CYC1*, with stable expression until day 4, followed by lower expression at subsequent time points. Expression of *PPARA* and *NRF1* transiently decreased after birth (Additional file 2: Figure S1B). The decrease in expression of *PPARGC1A*, *PPARGC1B* and *ERRA* is consistent with the decline in mitochondrial density.

Accordingly, based on ultra-structure, relative mtDNA measurements, expression and activity of key mitochondrial enzymes as well as expression of transcription factors and co-regulators controlling levels of mitochondrial factors, we concluded that mitochondrial density and function declined remarkably during the transition from BAT to WAT.

### Global gene expression analysis of postnatal brown to white adipose transformation

To obtain a global view of gene expression changes during the BAT to WAT transformation in the perirenal adipose depot, tag-based sequencing was performed on pools of mRNA from days −2, 0, 0.5, 1, 2, 4 and 14. The resulting reads were mapped to the October 2012 release of the sheep genome (v3.1) [17]. The library sizes obtained for the seven samples are approximately 7,400,000. Out of these, approximately 94% of the reads mapped to the reference genome, with approximately 56% of reads mapping uniquely to the reference genome (Additional file 3: Table S2). Nearly 40% of the reads, those mapping to multiple locations, were discarded. The uniquely mapped reads covered 13,963 out of 16,229 annotated genes in the sheep genome, with an average read depth of 16X. The relatively low number of reads mapping to gene-annotated regions (20%) accounts for the low annotation coverage available for the sheep genome. The number of mapped reads per annotated gene was counted, and normalized read counts per gene for the 7 time points were calculated (Additional file 4: Table S3). To facilitate downstream analyses, the human genes homologous to the sheep genes were mined, and the results from the gene expression analysis are discussed using the human protein symbols and names.

Next, we compared the expression of the genes measured by RT-qPCR in Figures 1, 2 and Additional file 2: Figure S1B to their expression in the tag-based sequencing data. Expression of *UCP1*, *DIO2*, *PPARGC1A*, *PPARGC1B*, *CS*, *CYC1* and *ERRA* decreased from day 0 to day 14 in both the sequencing data and when measured by RT-qPCR (Additional file 4: Table S3, Figures 1, 2 and Additional file 2: Figure S1B). In general, there was a relatively high correlation in the expression data obtained by the two methods.

The gene expression profiles were made from whole perirenal adipose tissue samples containing adipose as well as non-adipose cells. The amount and composition of non-adipose cells might have changed during the transformation of the tissue, thereby potentially influencing the differential gene expression between time points. However, the most highly expressed gene at both day 0 and day 14 was fatty acid-binding protein 4 (*FABP4*), a gene known to be strongly enriched in adipocytes (Additional file 4: Table S3). Among the 20 genes with the highest expression level at day 0 and day 14 were several genes encoding ribosomal proteins, *FABP5*, the fatty acid transporter cluster of differentiation 36 (*CD36*), the glycolytic enzyme aldolase A (*ALDOA*) and regulator of cell cycle (*RGCC*), a cell cycle regulator and kinase modulating protein. Genes highly expressed at day 14 included the pentose phosphate pathway enzyme transaldolase (*TALDO1*) and catalase (*CAT*). When comparing the 20 most highly expressed genes, genes related to fatty acid oxidation, electron transport chain and ATP synthase activity were more prevalent at day 0 compared to day 14.

### Identification of three phases in the brown to white adipose tissue transformation process

**Figure 3:**
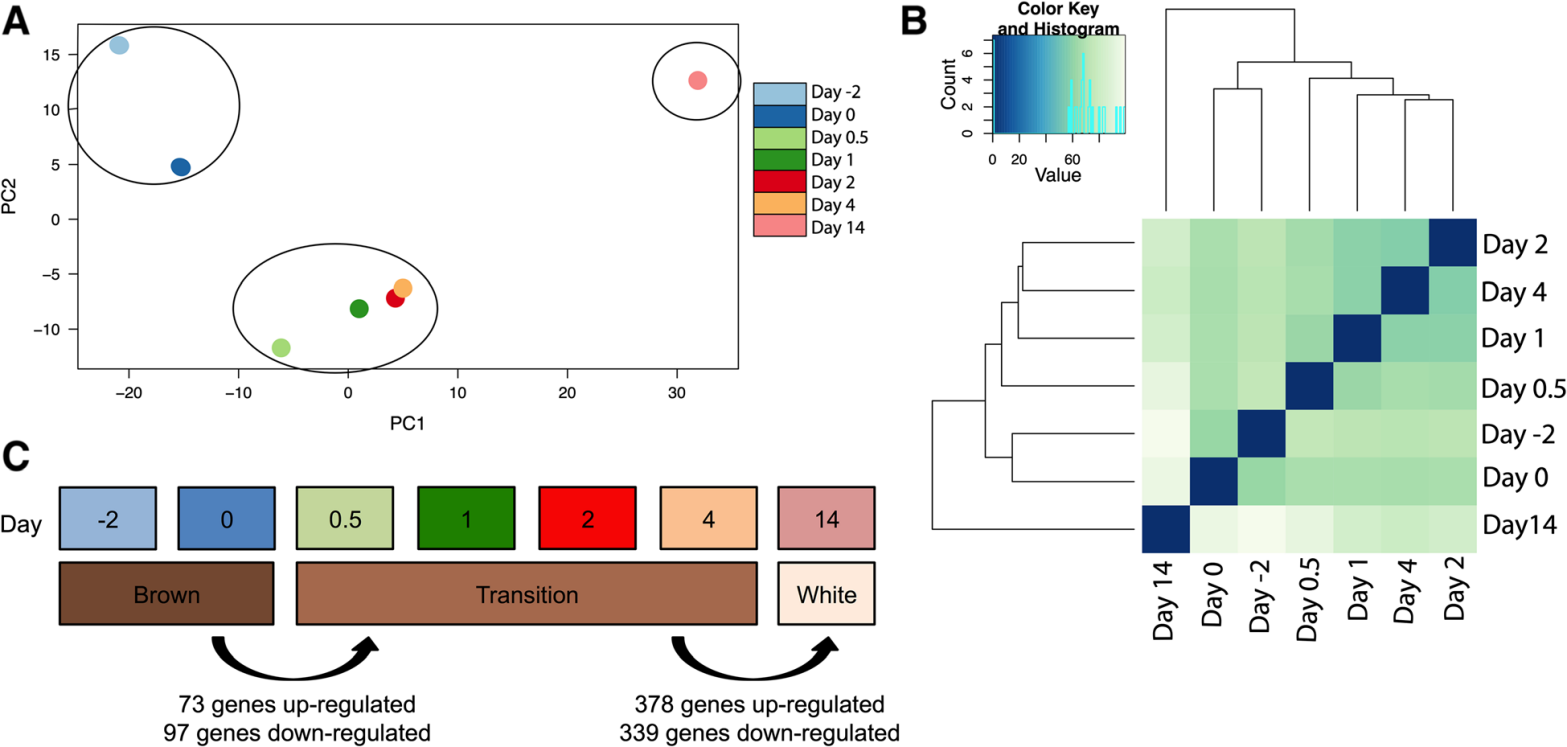
**Identification of the brown adipose phase, transition phase and white adipose phase. (A)** Principal component analysis (PCA) plot for the expression data from the seven time points showing the clustering of time points in the first two components. **(B)** Heatmap showing the hierarchical clustering based on Euclidean distances between the time points. **(C)** Allocation of the different time points to the three phases and summary of numbers of induced and repressed genes between phases.

To analyze the distribution of gene expression, a PCA was performed on the total gene expression data set (Figure 3A). The PCA plot indicated that total gene expression at the different time points clustered into three groups: a group including days −2 and 0, a second group including days 0.5, 1, 2 and 4, and a third group comprising day 14. Hierarchical clustering of the total gene expression data set clustered the 7 time points into the same three groups (Figure 3B). We interpreted the three clusters as distinct phases in the BAT to WAT transition (Figure 3C). At days −2 and 0 the tissue is in the brown adipose phase. During the phase composed of days 0.5, 1, 2 and 4 the tissue is expected to be in a thermogenic state in order to keep the lambs warm. At the same time points expression of some BAT-associated genes starts to drop, e.g. illustrated by the decrease in *PPARGC1A* expression from day 0 to day 0.5 (see Figure 1B). Therefore, we designate this period the transition phase. Day 14 represents the white adipose phase, as was also suggested by tissue morphology, mitochondrial numbers and function as well as expression level of *UCP1* (Figures 1 and 2).

**Figure 4:**
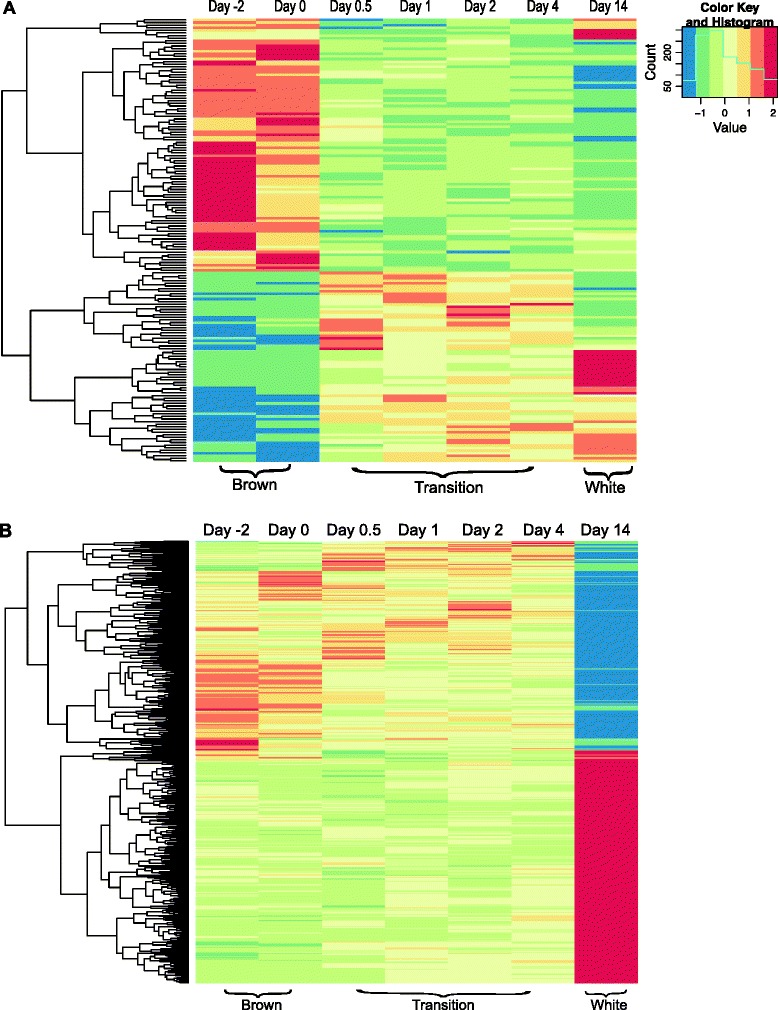
**Gene expression changes in the two phase shifts. (A)** Heatmap of the 170 genes differentially expressed from the brown adipose phase to the transition phase. **(B)** Heatmap of the 717 genes differentially expressed from the transition phase to the white adipose phase.

The expression of 170 genes changed significantly (*p*-value < 0.1) between the brown adipose phase and the transition phase (Additional file 5: Table S4). Of these, 73 genes were up-regulated and 97 genes were down-regulated (Figure 3C). A heatmap with Euclidian distances for the 170 genes is shown in Figure 4A. GO enrichment analysis (*p*-value < 10 e-5 and FDR < 0.5) of the 73 up-regulated genes revealed that they were enriched for genes related to “negative regulation of adaptive immune response” and “muscle cell migration”, whereas the 97 down-regulated genes were enriched for genes related to “organic acid metabolic processes” (Table 1).

**Table 1:** **GO enrichment analysis of genes differentially expressed from the brown adipose to the transition phase**

**GO term**	**Total number of genes in GO term**	**Number of differentially expressed genes**	***p*** **-value**
**Enrichment from up-regulated genes**			
Muscle cell migration	21	4	1.38E-06
Negative regulation of adaptive immune response	16	3	3.40E-05
**Enrichment from down-regulated genes**			
Organic acid metabolic process	722	16	1.26E-06
Isocitrate metabolic process	5	3	1.48E-06
Small molecule metabolic process	2003	28	1.03E-06

Between the transition phase and the white adipose phase, the expression of 717 genes changed significantly, of which 378 genes were up-regulated and 339 were down-regulated (Figure 3C and Additional file 6: Table S5). These differentially expressed genes are presented in a heatmap in Figure 4B. A GO enrichment analysis (*p*-value < 10 e-5 and FDR < 0.05) demonstrated that the 378 up-regulated genes were enriched for genes related to “cell death” and “negative regulation of cell death” (Table 2). The 339 down-regulated genes were enriched for genes related to “metabolic process”, including “fatty acid beta-oxidation” (Table 2).

**Table 2:** **GO enrichment analysis of genes differentially expressed from the transition to the white adipose phase**

**GO term**	**Total number of genes in GO term**	**Number of differentially expressed genes**	***p*** **-value**
**Enrichment from up-regulated genes**			
Enzyme linked receptor protein signaling pathway	819	38	3.11E-06
Negative regulation of cell death	877	36	7.75E-05
Cell death	2536	80	6.11E-05
**Enrichment from down-regulated genes**			
Generation of precursor metabolites and energy	361	67	9.08E-48
Oxidation-reduction process	306	49	1.21E-31
Cellular respiration	135	33	9.55E-28
Mitochondrial ATP synthesis coupled electron transport	46	19	2.16E-21
Metabolic process	9528	242	9.96E-15
Fatty acid beta-oxidation	35	11	2.26E-11
Lipid modification	129	13	8.84E-07
Translation	610	29	4.09E-06
Mitochondrion organization	276	18	4.64E-06
Monocarboxylic acid transport	88	9	3.86E-05

**Figure 5:**
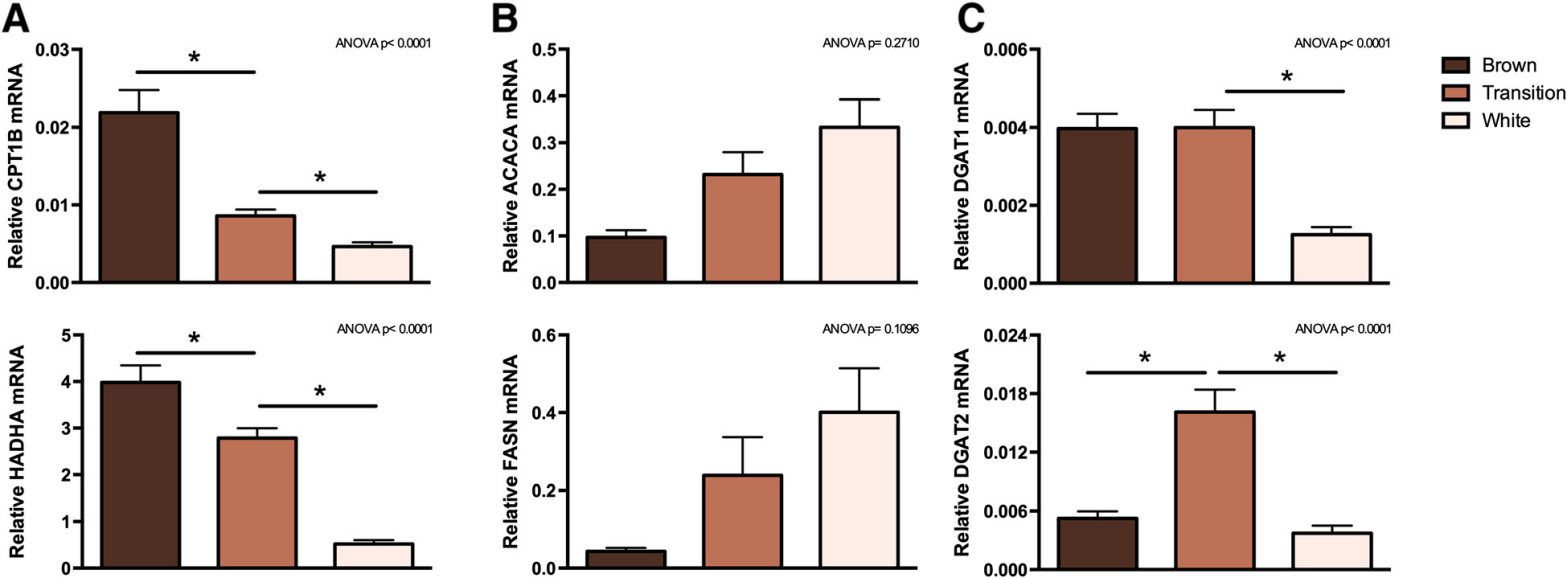
**Expression of selected metabolic enzymes related to fatty acid metabolism.** Total RNA was isolated from perirenal adipose tissue and used for RT-qPCR analysis. Relative expression was measured for: **(A)** carnitine palmitoyltransferase 1b (*CPT1B*) and hydroxyacyl-CoA dehydrogenase subunit α (*HADHA*); **(B)** acetyl-CoA carboxylase (*ACACA*) and fatty acid synthase (*FASN*); **(C)** diacylglycerol O-acyltransferase 1 (*DGAT1*) and *DGAT2*. The mRNA expression levels were normalized to expression of β-actin (*ACTB*). Data are mean + SEM (brown, n = 9; transition, n = 20; white, n = 15); *, *p* < 0.05.

The changes in gene expression related to the GO term “fatty acid beta-oxidation” were investigated in more detail by RT-qPCR (Figure 5). Of notice, the white adipose phase included samples from days 14, 30 and 60 for RT-qPCR measurements, whereas the white adipose phase for the global gene expression analysis included samples from day 14 only (see Figure 3). Two key enzymes in β-oxidation are carnitine palmitoyltransferase 1B (CPT1B) and the hydroxyacyl-CoA dehydrogenase complex (HADH). The relative mRNA expression levels of both *CPT1B* and the catalytic subunit α of HADH (*HADHA*) decreased from the brown adipose phase to the transition phase and from the transition phase to the white adipose phase (Figure 5A). We also measured expression of two genes involved in fatty acid synthesis by RT-qPCR: acetyl-CoA carboxylase 1 (*ACACA*) and fatty acid synthase (*FASN*). Expression of both tended to increase during the postnatal adipose transformation (Figure 5B), suggesting a higher rate of fatty acid synthesis in WAT compared to BAT.

The GO term “metabolic process” included 242 differentially expressed genes, a number of which have been measured by RT-qPCR, including *UCP1*, *CYC1* and *CS*. The two isoforms of the TAG synthesis enzymes diacylglycerol O-acyltransferase 1 (DGAT1) and DGAT2, were also among the regulated metabolic genes. RT-qPCR measurements confirmed a decreased expression of *DGAT1* and *DGAT2* in the white adipose phase compared to the transition phase (Figure 5C).

Of the 170 genes differentially expressed between the brown adipose and transition phase and 717 genes differentially expressed between the transition and the white adipose phase, 38 genes were in common. A Venn diagram of the 849 regulated genes is shown in Additional file 7: Figure S2. Fifteen of the 38 common genes were down-regulated at both phase shifts, whereas 9 genes were up-regulated at both phase shifts. Among the 15 consistently down-regulated genes were several mitochondrial genes, e.g. the TCA cycle enzyme isocitrate dehydrogenase 3α (*IDH3A*), and the two transcription factors myeloid leukemia factor 1 (*MLF1*) and autoimmune regulator (*AIRE*) (Additional file 8: Figure S3). Among the consistently up-regulated genes were two receptors involved in cellular lipid uptake: low density lipoprotein receptor-related protein 1 (*LRP1*) and macrophage scavenger receptor 1 (*MSR1*). The 38 genes also included 9 genes transiently up-regulated and 5 genes transiently down-regulated during the transition phase (Additional file 8: Figure S3). Among the genes up-regulated during the transition phase were two enzymes involved in TAG synthesis: the mitochondrial glycerol-3-phosphate acyltransferase (*GPAM*) and 1-acylglycerol-3-phosphate O-acyltransferase 9 (*AGPAT9*).

### Transcriptional components regulated between the three phases of adipose tissue transformation

Expression of 17 transcription factors and co-regulators was significantly changing between the brown adipose and the transition phase, of which 7 were up-regulated (Additional file 9: Table S6). Between the transition and the white adipose phase, 74 transcriptional components were differently expressed, with 48 being up-regulated (Additional file 9: Table S6). Four transcription factors exhibited differential expression at both phase shifts. Using *p* < 0.05 as the cut-off for adjusted *p*-value, two of the 17 transcriptional components, differently expressed between the brown adipose and the transition phase, had consensus putative response elements in an enriched set of the 170 genes displaying altered expression in the same phase shift. The two transcription factors were nuclear receptor subfamily 1, group H, member 3 (*NR1H3*, also called *LXRA*) and v-myc avian myelocytomatosis viral oncogene homolog (*MYC*). Of the 717 differently expressed genes between the transition and the white adipose phase, an enriched set of genes (adjusted *p-*value < 0.05) contained consensus putative response elements for six transcription factors that were themselves regulated in the same phase shift. The six transcription factors are *NR1H3*, *MYC*, B-cell lymphoma 6 (*BCL6*), estrogen receptor 1 (*ESR1*, also called *NR3A1* or *ESRA*), v-rel reticuloendotheliosis viral oncogene homolog A (*RELA*) and krüppel-like factor 4 (*KLF4*).

**Figure 6:**
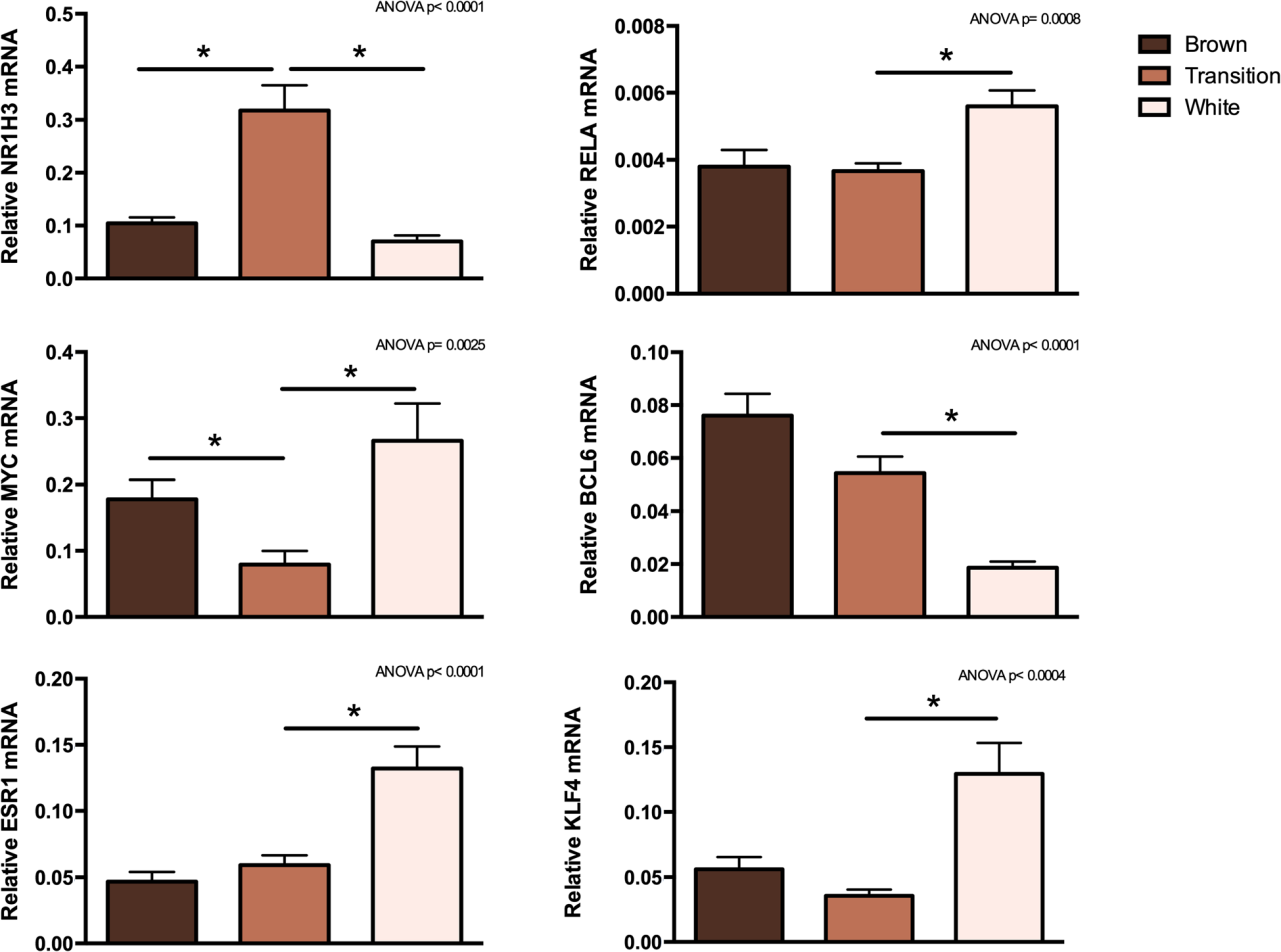
**Expression of regulated transcriptional components with consensus putative response elements in an enriched set of regulated genes.** Total RNA was isolated from perirenal adipose tissue and used for RT-qPCR analysis. Relative expression was measured for nuclear receptor subfamily 1, group H, member 3 (*NR1H3*), v-myc avian myelocytomatosis viral oncogene homolog (*MYC*), B-cell lymphoma 6 (*BCL6*), estrogen receptor 1 (*ESR1*), v-rel reticuloendotheliosis viral oncogene homolog A (*RELA*) and krüppel-like factor 4 (*KLF4*). The mRNA expression levels were normalized to expression of β-actin (*ACTB*). Data are mean + SEM (brown, n = 9; transition, n = 20; white, n = 15); *, *p* < 0.05.

The expression levels of these six transcription factors in the three phases were validated by RT-qPCR (Figure 6). Expression of *NR1H3* and *MYC* was significantly increased and decreased, respectively, in the transition phase compared to both the brown and the white adipose phase (Figure 6). Expression of the three transcription factors *ESR1*, *RELA* and *KLF4* was up-regulated between the transition and white adipose phase, whereas *BCL6* was significantly down-regulated from the transition to the white adipose phase (Figure 6).

**Figure 7:**
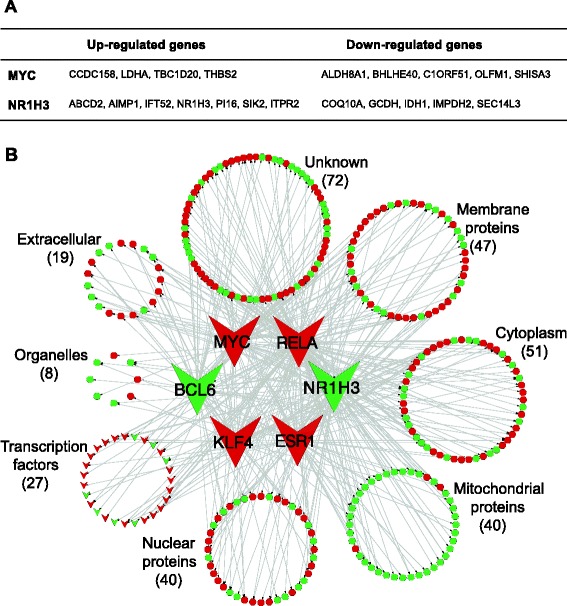
**Genes with altered expression that potentially are controlled by transcription factors regulated during the transformation. (A)** List of genes regulated from the brown adipose phase to the transition phase, which have consensus putative response elements for nuclear receptor subfamily 1, group H, member 3 (NR1H3) and v-myc avian myelocytomatosis viral oncogene homolog (MYC). **(B)** Subcellular localization of differentially expressed genes from the transition phase to the white adipose phase that have consensus putative response elements for NR1H3, MYC, B-cell lymphoma 6 (BCL6), estrogen receptor 1 (ESR1), krüppel-like factor 4 (KLF4) and v-rel reticuloendotheliosis viral oncogene homolog A (RELA). Green nodes indicate down-regulated genes in the white adipose phase as compared to the transition phase and red nodes indicate up-regulated genes in the white adipose phase as compared to the transition phase. The corresponding gene names are listed in Additional file 10: Table S7.

Figure 7A lists genes with differential expression between the brown adipose and transition phase that have a consensus putative response element for either *MYC* or *NR1H3*. Among the up-regulated genes that were potentially regulated by MYC from the brown adipose to the transition phase were the adhesion protein thrombospondin 2 (*THBS2*) and the Rab1 GTPase activator TBC1 domain family member 20 (*TBC1D20*). Among the down-regulated genes from the brown adipose phase to the transition phase potentially regulated by MYC were a 9-cis-retinoic acid synthesizing enzyme, aldehyde dehydrogenase 8 family member A1 (*ALDH8A1*) and the transcription factor basic helix-loop-helix family member E40 (*BHLHE40*).

Figure 7B depicts the subcellular distribution of the genes potentially regulated by MYC, ESR1, RELA, BCL6, KLF4 or NR1H3 between the transition and the white adipose phase. Gene names corresponding to Figure 7B are presented in Additional file 10: Table S7. Forty of the regulated genes were mitochondrial genes, 37 of which were down-regulated. This is in accordance with the decreased activity and amount of mitochondria in the white adipose phase (Figure 2 and Additional file 2: Figure S1). Half of the down-regulated mitochondrial genes have been described to be regulated by RELA. Nineteen genes encoded secreted proteins, 11 of which were up-regulated, including two pro-angiogenic factors: vascular endothelial growth factor B (*VEGFB*) and angiopoietin-related protein 2 (*ANGPTL2*), and one anti-angiogenic factor, serpin peptidase inhibitor F1 (*SERPINF1*) (Additional file 10: Table S7).

### Expression of white, brite/beige and brown adipose markers in the three phases of adipose tissue transformation

**Figure 8:**
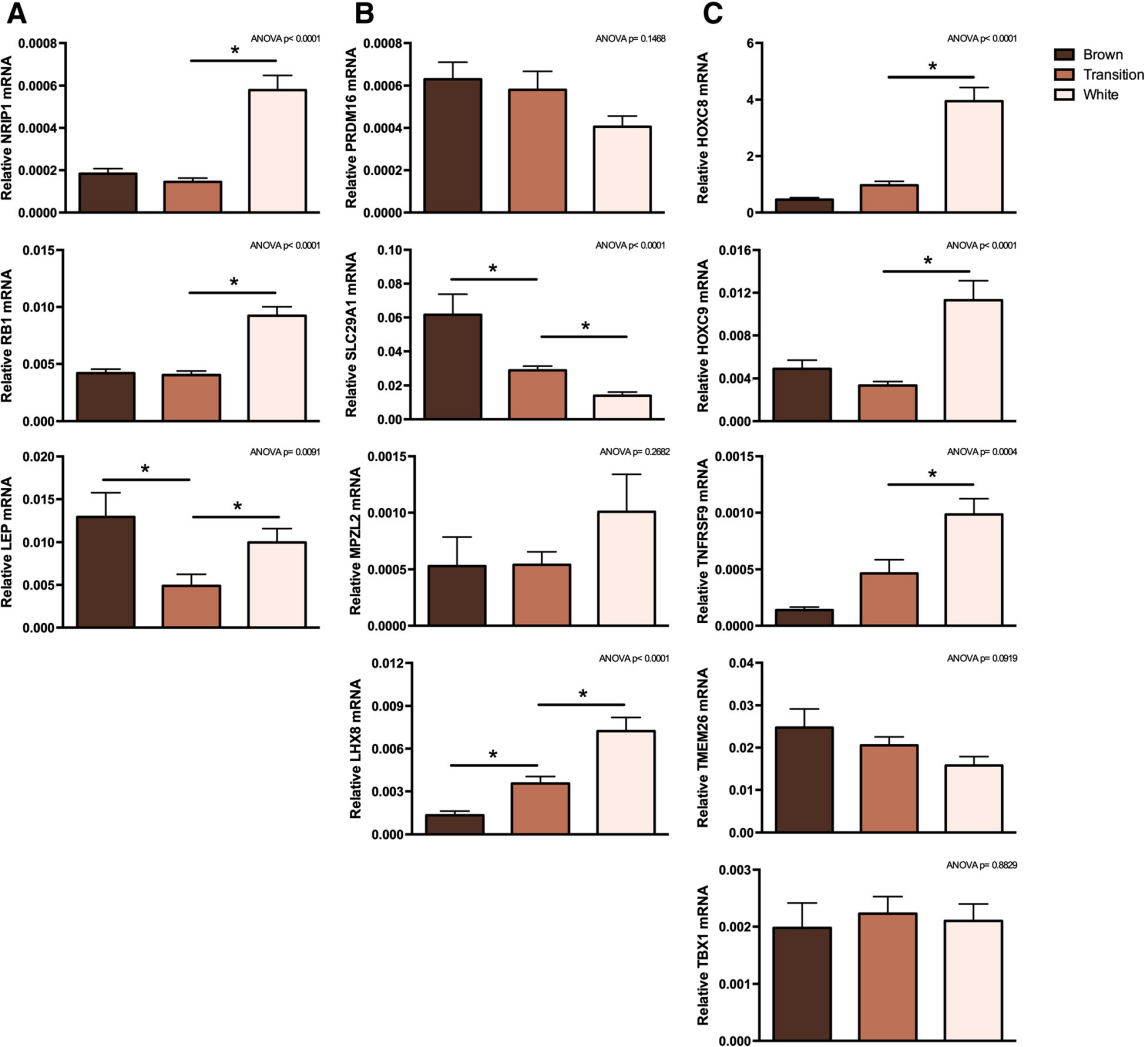
**Expression of genetic markers for white, brown and brite/beige adipose tissue during the three phases of the transformation.** Gene expression was determined by RT-qPCR. **(A)** Relative levels of genes associated with white adipocytes: nuclear receptor-interacting protein 1 (*NRIP1*), retinoblastoma 1 (*RB1*) and leptin (*LEP*). **(B)** Relative levels of the brown adipose associated and marker genes: PR domain containing 16 (*PRDM16*), solute carrier family 29 member 1 (*SLC29A1*), LIM homeobox 8 (*LHX8*) and myelin protein zero-like 2 (*MPZL2*). Zinc finger protein 1 (*ZIC1*) was not detectable in any of the adipose samples. **(C)** Relative levels of the brite/beige adipose markers: homeobox C8 (*HOXC8*), *HOXC9*, tumor necrosis factor receptor superfamily member 9 (*TNFRSF9*), transmembrane protein 26 (*TMEM26*) and T-box 1 (*TBX1*). The mRNA expression levels were normalized to expression of β-actin (*ACTB*). Data are mean + SEM (brown, n = 9; transition, n = 20; white, n = 15); *, *p* < 0.05.

To study the three phases in the transformation process in more detail, we measured a number of brown and white adipose marker genes by RT-qPCR. As evident from Figure 1, we observed a down-regulation of *UCP1* between the transition and white adipose phase, and a stepwise decrease in expression of *DIO2* and *PPARGC1A* through the three phases of the transformation (Additional file 11: Figure S4). Expression of two transcriptional co-regulators promoting white adipogenesis: nuclear receptor-interacting protein 1 (*NRIP1*, also called *RIP140*) and retinoblastoma 1 (*RB1*), was increased between the transition and the white adipose phase (Figure 8A). A typical white adipose marker gene leptin (*LEP*) displayed decreased expression in the transition phase compared to both the brown and white adipose phase (Figure 8A). Expression of the key transcriptional driver of brown adipogenesis, PR domain containing 16 (*PRDM16*), was not changed significantly between the phases (Figure 8B). Overall, these measurements supported the brown to white adipose transformation occurring in the sheep perirenal adipose tissue within the first two weeks after birth.

To address if the sheep perirenal adipose tissue qualified as being brown, brite/beige or a mixture of brown and brite/beige at birth, and whether this status of the tissue changed over time, we measured a number of recently proposed marker genes selectively expressed in brown versus brite/beige adipose tissue and adipocytes [4,31-34]. We determined the expression of the classical brown adipose marker genes solute carrier family 29 member 1 (*SLC29A1*), LIM homeobox 8 (*LHX8*), myelin protein zero-like 2 (*MPZL2*, also called *EVA1*) and zinc finger of the cerebellum 1 (*ZIC1*). *SLC29A1* was expressed at birth and elicited a stepwise down-regulation through the two adipose phase shifts (Figure 8B). *MPZL2* expression did not significantly change, whereas the *LHX8* mRNA increased steadily through the three phases (Figure 8B). *ZIC1* was not detectable in any of the perirenal adipose samples, but was easily detectable in sheep brain (data not shown). We also measured the expression of the three brite/beige marker genes homeobox C8 (*HOXC8*), *HOXC9* and tumor necrosis factor receptor superfamily member 9 (*TNFRSF9*, also called *CD137*). *HOXC8* and *HOXC9* are marker genes for both WAT and brite/beige adipose tissue [31,32]. The expression of all three genes increased from the transition phase to the white adipose phase (Figure 8C). However, expression of the brite/beige marker genes transmembrane protein 26 (*TMEM26*) and T-box protein 1 (*TBX1*) did not change (Figure 8C). In summary, three out of four markers of classical BAT were detectable, and two out of these three changed expression over time. All five measured brite/beige markers were detectable: the expression of three increased and two remained unchanged from the transition to the white adipose phase. Thus, markers of both brown and brite/beige adipose tissue were expressed in perirenal adipose tissue from sheep, and most of these markers displayed altered expression over time.

## Discussion

Plasticity of adipose tissues is important for adaptation to changing physiological conditions [35]. In response to prolonged cold exposure, subcutaneous WAT depots of rodents undergo a transformation process during which numerous brite/beige adipocytes appear, thereby increasing overall thermogenic capacity of the animal [6,7,35]. In large mammals, a substantial part of BAT present in the newborn converts to WAT after birth, which may reflect a diminished need for endogenous thermogenesis after the early postnatal period. As little molecular insight into this conversion in large mammals is available, we have in the present study conducted a detailed analysis of the postnatal transformation of perirenal adipose tissue in sheep. We chose this particular tissue, as it is the most frequently studied BAT depot in large mammals. Moreover, we reasoned that the transformation of this depot was suitably modeling the postnatal brown to white adipose transformation in humans. The perirenal adipose depot from adult humans has recently been characterized at the morphological and transcriptional level [36]. This characterization revealed an enormous variation in expression levels of *UCP1*.

The postnatal transformation process from BAT to WAT in sheep perirenal adipose tissue occurred within the first two weeks after birth as determined by changes in tissue morphology, gene expression and mitochondrial density. Adipocyte morphology changed from being mainly multilocular to unilocular and the amount of mitochondria decreased. The expression of brown adipocyte-selective genes, e.g. *UCP1*, *DIO2* and *PPARGC1A*, declined, as did expression of additional genes encoding mitochondrial proteins. To understand the adipose transformation in more detail, we performed a global gene expression analysis with 7 time points ranging from approximately two days before birth to two weeks after birth. By two independent analyses of the gene expression data, we determined that the transformation clustered into three phases: a brown adipose phase, a transition phase and a white adipose phase.

### Regulated transcription factors and co-regulators

Between the brown adipose and the transition phase were 170 genes differentially expressed, including 17 transcriptional regulators, 10 of which were down-regulated (Additional file 9: Table S6). Five of these have chromatin modifying activity: circadian locomotor output cycles kaput (*CLOCK*), nuclear receptor co-activator 1 (*NCOA1*, also called *SRC1*), proviral insertion site in Moloney murine leukemia virus lymphomagenesis (*PIM1*), and the SWI/SNF-related matrix-associated actin-dependent regulator of chromatin subfamily members *SMARCC2* and *SMARCD3*. This leaves open the possibility that extensive remodeling of chromatin is occurring between the brown adipose and the transition phase. In accordance with its down-regulation in the perirenal adipose transformation (Additional file 9: Table S6), NCOA1 has been reported to promote BAT activity in mice [37].

Between the transition and the white adipose phase 717 genes were differentially expressed, of which 74 were transcription factors or co-regulators (Additional file 9: Table S6). The list of regulated transcriptional regulators included a few factors known to be differently expressed in mouse WAT and BAT, e.g. *NRIP1* and cell death-inducing DFFA-like effector a (*CIDEA*). Among the down-regulated transcriptional regulators from the transition to the white adipose phase were some related to brown adipocyte function: early B-cell factor 2 (*EBF2*), which have been described to induce expression of brown adipose-specific PPARG target genes [38], leucine-rich PPR motif-containing protein (*LRPPRC*), a PPARGC1A co-activator playing an important role in BAT differentiation and function [39] and Y box binding protein 1 (*YBX1*), an inducer of bone morphogenetic protein 7 (*BMP7*) transcription and brown adipocyte differentiation [40].

### Regulated transcription factors with a consensus putative response element in an enriched set of differentially expressed genes

Expression of four transcription factors *NR1H3*, *MYC*, *AIRE* and *MLF1* was regulated at both phase shifts. The former two have a consensus putative response element in an enriched set of genes displaying altered expression at the two phase shifts. Expression of *NR1H3* and *MYC* was transiently increased and decreased, respectively, during the transition phase (Figure 6). Consistent with the opposite regulation during the transition phase, NR1H3 have been reported to suppress *MYC* expression in colon cancer cells [41] (Additional file 12: Figure S5). MYC is known to inhibit adipogenesis [42,43], which might explain why it is down-regulated in the transition phase, which includes tissue expansion. NR1H3 has been described to regulate gene expression linked to several important aspects of both brown and white adipocyte biology, including adipogenesis, energy expenditure, lipolysis and glucose transport [44]. NR1H3 was reported to be present at higher levels in mouse BAT than WAT [45] and to suppress PPARγ-induced *UCP1* expression by binding to the *UCP1* enhancer together with NRIP1 in mouse adipocytes [46]. Accordingly, NR1H3 can regulate the expression of brown adipocyte-selective genes. However, the increased *NR1H3* expression in the transition phase did not correlate with decreased *UCP1* expression in our study, which might be explained by the relative low expression of *NRIP1* in the transition phase (Figure 8).

Beside *NR1H3* and *MYC*, four other transcription factors, *RELA*, *KLF4*, *ESR1* and *BCL6*, were regulated between the transition and the white adipose phase and found to have consensus putative response elements in an enriched number of genes regulated between these two phases. The former three were up-regulated from the transition to the white adipose phase, whereas *BCL6* was down-regulated (Figure 6). Both RELA and ESR1 have been described to stimulate *MYC* expression [47,48], which would be consistent with the increased expression of *MYC* in the white adipose phase (Figure 6 and Additional file 12: Figure S5). Of interest, RELA and ESR1 have opposite effects on adipogenesis, as both knockdown of *RELA* and activation of ESR1 by estrogen supplementation attenuated adipogenesis [49,50]. WAT from mice with an adipocyte-specific knockout of *RELA* have decreased lipid droplet size, increased glucose uptake and reduced expression of adipogenic marker genes such as *LEP*, *PPARG* and adiponectin (*ADIPOQ)*. Furthermore, the mice are obesity resistant and less sensitive to cold [49] (Additional file 12: Figure S5). *ESR1* knockout mice have increased fat mass caused by adipocyte hyperplasia [51]. In addition, ESR1 can inhibit the transcriptional activity of RELA [52] (Additional file 12: Figure S5). Based on this, we speculate that RELA and ESR1 might contribute to the regulation of TAG accretion during the adipose transformation. RELA might also contribute to the observed mitochondrial depletion, as RELA negatively impacts mitochondrial content in C2C12 myocytes [53]. RELA has putative response elements in the promoter of 20 genes encoding mitochondrial proteins regulated between the transition and the white adipose phase, of these 17 were down-regulated. This is in accordance with RELA functioning both as a transcriptional activator and repressor [54].

RELA has been described to repress *BCL6* expression through interferon regulatory factor 4 [55]. This is in accordance with the increased expression of *RELA* and the decreased expression of *BCL6* in the white adipose phase (Figure 6). BCL6 is a transcriptional repressor with the ability to reduce the expression of e.g. *MYC* [56] (Additional file 12: Figure S5). The expression of *BCL6* is strongly down-regulated by growth hormone in 3T3-F442A adipocytes [57]. Apart from this, BCL6 has not been linked to adipocyte or adipose tissue function. KLF4 and RELA have been reported to be functionally intertwined, as they directly interact to induce expression of selected genes [58], but also to compete for interaction with a co-activator, thereby inhibiting each other’s activity [59] (Additional file 12: Figure S5). KLF4 is important for induction of adipogenesis *in vitro* and its expression was reported to be induced in pre-adipocytes by cAMP. KLF4 stimulates CCAAT/enhancer-binding protein β (*C/EBPB*) expression, and C/EBPB in turn down-regulates *KLF4* expression, thereby forming a negative feedback loop [60].

In summary, six transcription factors with differential expression during the adipose transformation have consensus putative response elements in an enriched set of the regulated genes, suggesting that they are involved in the control of the overall gene expression changes and thus potentially have an impact on remodeling of the tissue. Moreover, the six factors are mutually functionally linked, leaving open the possibility that they are part of a transcriptional network (Additional file 12: Figure S5).

### Brown and brite/beige markers

A number of marker genes selectively expressed in white, brite/beige and brown adipose tissue and adipocytes have been reported [4,31-34]. Some of these marker genes are not identified consistently between studies. Moreover, some markers have been identified from gene expression profiling of whole tissue samples, whereby non-adipose cells cannot be excluded to be a significant source of expression [31,34,61]. It is being discussed whether human BAT is composed of brown or brite/beige adipocytes or a mixture of these. Moreover, it is not fully established to what extent expression of white, brite/beige and brown adipose marker genes changes in a particular adipose depot during development or remodeling. To elucidate this in sheep, we analyzed the expression of selected marker genes in the brown adipose, transition and white adipose phase of the perirenal adipose depot. Although we only measured marker gene expression in the perirenal depot, and accordingly have not compared expression levels to those in other adipose depots, we could detect both brown (e.g. *LHX8*) and brite/beige (e.g. *TNFRSF9*) adipocyte marker genes in the newborn sheep (Figure 8). Co-expression of marker genes selective for brown and brite/beige adipose tissues have also been observed in a recent study of the human supraclavicular BAT depot [61]. In this human study, expression of *UCP1* in supraclavicular biopsies was positively associated with expression of both BAT markers (e.g. *ZIC1* and *LHX8*) and brite/beige adipose markers (e.g. *TBX1* and *TMEM26*), whereas expression of the two WAT and brite/beige adipose markers *HOXC8* and *HOXC9* correlated with low *UCP1* expression [61]. In our time course study, we did not observe a correlation between high *UCP1* expression and high expression of *ZIC1* (which was not detected), *LHX8*, *TBX1* or *TMEM26*, but we did detect higher expression of *HOXC8* and *HOXC9* in the white adipose state, where *UCP1* expression was low (Figure 8 and Additional file 11: Figure S4). A similar *HOXC9* profile in sheep perirenal adipose tissue has been reported by others [11].

BAT, brite/beige adipose and WAT marker gene expression have not previously been studied in detail during adipose tissue remodeling in large mammals. Based on the selective expression profile in mice, our observation that *HOXC8* and *HOXC9* are induced in the white adipose phase suggests that the perirenal adipose depot converts from brown (not brite/beige) to white (Figure 8). The brown adipose origin of the perirenal depot might be supported by the down-regulation of the BAT marker gene *SLC29A1* during whitening. However, the 5-fold increase in expression of *LHX8* from the brown to the white adipose phase and the absence of *ZIC1* expression were not consistent with this model. In addition to *HOXC8* and *HOXC9*, a number of other brite/beige marker genes were expressed shortly after birth (Figure 8). The expression of these was either unchanged or up-regulated during whitening. In summary, markers of both brite/beige and brown adipose tissue are expressed in the sheep perirenal adipose tissue at birth and the expression of a number of these changes substantially over time. The latter might be important to consider when analyzing adipose tissue type-selective gene expression.

### Model of the transformation process

The postnatal transformation from brown (or brite/beige) to white in the perirenal adipose tissue can occur through at least three different mechanisms: 1) through transdifferentiation of brown (or brite/beige) to white adipocytes; 2) through proliferation and differentiation of white adipogenic precursor cells and death of mature brown (or brite/beige) adipocytes; 3) through a combination of the two. In mice, brite/beige adipocytes can transdifferentiate into white adipocytes [34], but whether this observation extends to large mammals is unclear. If the white adipocytes arise exclusively from proliferation and differentiation of precursor cells, it would require an enormous cell turnover during the transition phase of the transformation, including extensive death of mature brown (or brite/beige) adipocytes. We would expect this to result in induction of cell cycle genes, but we did not observe this in the GO term analysis. Moreover, the expression profile of key cyclins (*CCNA*, *CCNB*, *CCND* and *CCNE*) was not consistent with the hypothesis of massive cell cycling during the transition phase (Additional file 4: Table S3). However, we cannot rule out that a small population of proliferating white adipose precursor cells contributed to the whitening of the tissue. In the shift from transition phase to white adipose phase, we did observe an up-regulation of genes related to cell death, but nearly half of the up-regulated cell death associated genes are negative regulators of cell death (Table 2). Thus, it is not obvious from the gene expression data if cell death is increased or not. Furthermore, we did not observe evidence for significant cell death in any of the tissue sections analyzed. Consistently, Lomax et al. [10] failed to detect apoptotic cells during the transformation of the perirenal adipose tissue in sheep.

An alternative to the transdifferentiation model is that the white adipocytes present in the white adipose phase are in fact masked brite/beige adipocytes, possibly of a different lineage than white adipocytes. These cells might have the potential to return to a thermogenic state, which would be compatible with existence of such masked adipocytes in mice [34]. Arguing against this scenario in sheep is that after postnatal day 5, *UCP1* and other thermogenic genes are no longer inducible in perirenal adipose tissue in response to repeated injections of a β-adrenergic agonist [10]. In summary, we consider it plausible that transdifferentiation of brown (or brite/beige) to white adipocytes is a significant component of the postnatal transformation of the perirenal adipose depot in sheep. Clearly, additional studies are required to validate to what extent transdifferentiation takes place, including a time course with more time points and a dedicated search for evidence of cell proliferation and death.

## Conclusions

By global gene expression profiling, we provide novel information of the postnatal BAT to WAT transformation in sheep. This transformation process is poorly understood in molecular terms, but is of significant interest, as a similar transformation occurs in human infants after birth. An improved understanding of this tissue remodeling increases insight into adipose plasticity and might allow identification of targets suitable for interfering with the balance between energy-storing and energy-dissipating adipose tissue. Our results reveal novel transcription factors linked to the adipose transformation process in large mammals. Clearly, validation of their relevance in adipose function will require dedicated functional studies. Finally, we show that expression of adipose tissue-type selective marker genes change substantially over time, which might be an underappreciated variable in such analyses.

## Additional files

Additional file 1: Table S1. Primers used for qPCR. Additional file 2: Figure S1. Transmission electron microscopy and expression of mitochondria-related genes during brown to white adipose transformation. Transmission electron microscopy and expression of mitochondria-related genes during brown to white adipose transformation. (A) Transmission electron micrographs of perirenal adipose tissue at postnatal days 0, 2, 4 and 14. Representative images are shown for each of the indicated time points (n = 3) in three different magnifications. (B) Total RNA was isolated from perirenal adipose tissue and used for RT-qPCR analysis. Relative expression was measured for peroxisome proliferator-activated receptor γ (PPARG) co-activator 1β (*PPARGC1B*), *PPARA*, estrogen-related receptor α (*ERRA*) and nuclear respiratory factor 1 (*NRF1*). The mRNA expression levels were normalized to expression of β-actin (*ACTB*). Data are mean + SEM (n =4-5); *, *p* < 0.05 vs. day 0. Additional file 3: Table S2. Read statistics for the sequencing data when mapped and annotated to the sheep genome. A table listing the counts of raw reads from the 7 time points, reads mapped to the sheep genome, uniquely mapped reads and reads mapped to 13,963 annotated genes in the sheep genome. The table also lists the percentage of total reads in the above mentioned classes. Additional file 4: Table S3. Normalized counts of genes from perirenal adipose tissue at postnatal days −2, 0, 0.5, 1, 2, 4 and 14. Additional file 5: Table S4. List of the 170 differentially expressed genes between the brown adipose phase and the transition phase. Additional file 6: Table S5. List of the 717 differentially expressed genes between the transition phase and the white adipose phase. Additional file 7: Figure S2. Venn diagram of differentially expressed genes between the three phases. Additional file 8: Figure S3. Heatmap of the 38 genes regulated between the three phases. Heatmap of the 38 genes regulated between the brown adipose phase and the transition phase as well as between the transition phase and the white adipose phase. Transcription factors are highlighted in yellow. Additional file 9: Table S6. Transcriptional regulators with changed expression levels between the three phases. Transcription factors and co-regulators with changed expression levels in the brown adipose to transition phase shift or in the transition to white adipose phase shift. Additional file 10: Table S7. List of differentially expressed genes from the transition phase to the white adipose phase that have consensus putative response elements for NR1H3, MYC, BCL6, ESR1, KLF4 or RELA. List of differentially expressed genes from the transition phase to the white adipose phase that have consensus putative response elements for nuclear receptor subfamily 1, group H, member 3 (NR1H3), v-myc avian myelocytomatosis viral oncogene homolog (MYC), B-cell lymphoma 6 (BCL6), estrogen receptor 1 (ESR1), krüppel-like factor 4 (KLF4) or v-rel reticuloendotheliosis viral oncogene homolog A (RELA). Genes are organized according to the subcellular localization of their encoded product. Additional file 11: Figure S4. Relative levels of brown adipose-associated genes during the three phases of the postnatal adipose transformation. Relative levels of brown adipose-associated genes during the three phases of the postnatal adipose transformation. These data are an alternative presentation of the data presented in Figure 1B. Total RNA was isolated from perirenal adipose tissue and used for RT-qPCR analysis. Relative expression was measured for uncoupling protein 1 (*UCP1*), type II iodothyronine deiodinase (*DIO2*) and peroxisome proliferator-activated receptor γ (PPARG) co-activator 1α (*PPARGC1A*). The mRNA expression levels were normalized to expression of β-actin (*ACTB*). Data are mean + SEM (brown, n = 9; transition, n = 20; white, n = 15); *, *p* < 0.05. Additional file 12: Figure S5. Mutual interactions between differentially expressed transcription factors with putative response elements in an enriched set of regulated genes. Mutual interactions between the six differentially expressed transcription factors with putative response elements in an enriched set of genes with altered expression during the adipose transformation. The brown oval-shaped circles represent the differentially expressed transcription factors; white oval-shaped circles represent other transcriptional components functionally linked to the differentially expressed transcription factors; stop arrows indicate repression/inhibition; arrows indicate activation/stimulation.

## Abbreviations

ACACA: Acetyl-CoA carboxylase 1
AGPAT9: 1-acylglycerol-3-phosphate O-acyltransferase 9
AIRE: Autoimmune regulator
ALDH8A1: Aldehyde dehydrogenase 8 family member A1
ALDOA: Glycolytic enzyme aldolase A
ANGPTL2: angiopoietin-related protein 2
ATP5B: ATP synthase β
BAT: Brown adipose tissue
BCL6: B-cell lymphoma 6
BHLHE40: Basic helix-loop-helix family member E40
BMP7: Bone morphogenetic protein 7
C/EBPB: CCAAT/enhancer-binding protein β
CAT: Catalase
CCN: Cyclin
CD36: Cluster of differentiation 36
CIDEA: Cell death-inducing DFFA-like effector a
CLOCK: Circadian locomotor output cycles kaput
CPT1B: Carnitine palmitoyltransferase 1B
CS: Citrate synthase
CYC1: Cytochrome c1
DGAT: Diacylglycerol O-acyltransferase
DIO2: Type II iodothyronine deiodinase
EBF2: Early B-cell factor 2
EDTA: Ethylenediaminetetraacetic acid
EPAS1: Endothelial PAS domain-containing protein 1
ERRA: Estrogen-related receptor α
ESR1: Estrogen receptor 1
FABP4: Fatty acid-binding protein 4
FASN: Fatty acid synthase
FDR: False discovery rate
GPAM: Mitochondrial glycerol-3-phosphate acyltransferase
HADH: Hydroxyacyl-CoA dehydrogenase complex
HADHA: HADH catalytic subunit α
HGNC: HUGO Gene Nomenclature Committee
HE: Hematoxylin-eosin
HOXC8: Homeobox C8
HUGO: Human Genome Organization
IDH3A: Isocitrate dehydrogenase 3α
KLF4: Krüppel-like factor 4
LEP: Leptin
LHX8: LIM homeobox 8
LRP1: Low density lipoprotein receptor-related protein 1
LRPPRC: Leucine-rich PPR motif-containing protein
MLF1: Myeloid leukemia factor 1
MPZL2: Myelin protein zero-like 2
MSR1: Macrophage scavenger receptor 1
MT-CO1: Mitochondrially encoded cytochrome c oxidase I
mtDNA: Mitochondrial DNA
MYC: V-myc avian myelocytomatosis viral oncogene homolog
NCOA1: Nuclear receptor co-activator 1
nDNA: nuclear DNA
NR1H3: Nuclear receptor subfamily 1, group H, member 3
NRF1: Nuclear respiratory factor 1
NRIP1: Nuclear receptor-interacting protein 1
PCA: Principal component analysis
PIM1: Proviral insertion site in Moloney murine leukemia virus lymphomagenesis
PPARG: Peroxisome proliferator-activated receptor γ
PPARGC1A: PPARG co-activator 1α
PRDM16: PR domain containing 16
RB1: Retinoblastoma 1
RELA: V-rel reticuloendotheliosis viral oncogene homolog A
RGCC: Regulator of cell cycle
RT-qPCR: Reverse transcription-quantitative polymerase chain reaction
SERPINF1: Serpin peptidase inhibitor F1
SLC29A1: Solute carrier family 29 member 1
SMARCC2: SWI/SNF-related matrix-associated actin-dependent regulator of chromatin subfamily member C2
ST7: Suppression of tumorigenicity 7
TAG: Triacylglycerol
TALDO1: Transaldolase
TBX1: T-box protein 1
TCA: Tricarboxylic acid
TEM: Transmission electron microscopy
TFIIB: Transcription factor IIB
TMEM26: Transmembrane protein 26
TNFRSF9: Tumor necrosis factor receptor superfamily member 9
UCP1: Uncoupling protein 1
VEGFB: Vascular endothelial growth factor B
WAT: White adipose tissue
YBX1: Y box binding protein 1
ZIC1: Zinc finger of the cerebellum 1
