# Supplementary material for: Global gene expression profiling of brown to white adipose tissue transformation in sheep reveals novel transcriptional components linked to adipose remodeling
Source: BMC Genomics. 2015 Mar 19;16(1):215. doi: 10.1186/s12864-015-1405-8 (PMC4407871; doi:10.1186/s12864-015-1405-8)
Supplement: Additional file 1: Table S1. — Primers used for qPCR. [file 12864_2015_1405_MOESM1_ESM.docx]

**Additional file 1: Table S1.** Primers used for qPCR.

| Gene | Amplicon length | Forward primers | Reverse primers |
| --- | --- | --- | --- |
| ACACA | **101** | TGGGGTTATTTCAGTGTTGCT | ATTGCTTCCTCTCGGTTTTCT |
|  |  |  |  |
| ACTB | **149** | CAGACAGGATGCAGAAAGAGA | CTTGCTGATCCACATCTGCT |
|  |  |  |  |
| BCL6 | **103** | CGAGTCCCTTTTCGGAATCT | GCCAGCTAGGATTTGCACTA |
|  |  |  |  |
| CPT1B | **143** | GATCTATCTGTCTGGGGTCAGG | CCACGTTGTAGCAGGAGGA |
|  |  |  |  |
| CS | **94** | CCACTAATCTACACCCCATGTC | TTGACACCCTCTGAATATGCTC |
|  |  |  |  |
| CYC1 | **85** | CAGCTCCCACTACGGACAC | GCGCTGACAGAATCACTTTC |
|  |  |  |  |
| DGAT1 | **109** | TCTACACCATCCTCTTCCTCAA | GCCTTCTTACCTGCCAAAG |
|  |  |  |  |
| DGAT2 | **98** | CAAGGGCTTTGTGAAACTGG | CCTCAAAGATCACCTGCTTGT |
|  |  |  |  |
| DIO2 | **72** | AGGAAGAGGCAGTTGGAGAAA | GGCATCCTCAGCGTAGACTT |
|  |  |  |  |
| ESRRA | **118** | CTATGGTGTGGCATCCTGTGA | CTTGCGTCTCCGCTTGGT |
|  |  |  |  |
| ESR1 | **77** | AAGTGCTATGAAGTGGGCAT | CGCTTGTGCTTCAACATTCT |
|  |  |  |  |
| FASN | **112** | CTCATTCACTCGGGTTCTGG | GGAGGTATGCCCGCTTTT |
|  |  |  |  |
| HADHA | **79** | TGGGTATACCTGCTGCTTTTG | ACCAGTCCCATTTTCTTTGCT |
|  |  |  |  |
| HOXC8 | **66** | GTAATGCGGTTTTGTTGTTGTT | GCAGTCTCAGTTGTTGGGTTT |
|  |  |  |  |
| HOXC9 | **126** | TGGACTCGCTCATCTCTCAC | TACAGGACGGAAAATCGCTAC |
|  |  |  |  |
| KLF4 | **80** | ATGGGCAAGTTCGTGTTGAA | TTGCTAACACTGATGACCGA |
|  |  |  |  |
| LEP | **59** | CCTCTCCTGAGTTTGTCCAAG | GCGAGGATCTGTTGGTAGATT |
|  |  |  |  |
| LHX8 | **162** | AATGGGATTAGTGTTGAAGGC | CAGTTTCTGGAGAGTCTGTGC |
|  |  |  |  |
| MPZL2 | **70** | GGCAGGGTTTTGTTTTTGTT | GTGGTTTTGGCTTGTGCTACT |
|  |  |  |  |
| MT-CO1[[1](#_ENREF_1)] | **115** | ATCGGTGGATTCGGCAACTGA | GGAGTAACAGGAAAGATGGGGGAAG |
|  |  |  |  |
| MYC | **98** | GACACGGAGGAGAATGACAA | ATCTGGTCACGAAGAGCAAA |
|  |  |  |  |
| NR1H3 | **138** | TGCAGGAGATCGTGGATTTT | CACTTCCAGGGTTGTACCTC |
|  |  |  |  |
| NRF1 | **144** | TCCAAACCCAACCCTGTCT | CAGCTCTGAGTTAACCTCCTGT |
|  |  |  |  |
| NRIP1 | **156** | GCAGTGTGACCAACAGCAAG | CAAGTGCTCAGGGAAAGGAG |
|  |  |  |  |
| PPARGC1A | **109** | GGAACAGCAGCAGAGACAAAT | TCTGGGGTCAGAGGAAGAGAT |
|  |  |  |  |
| PPARGC1B | **95** | CAGTGGTGCCCAGAGAACT | AGGGCCTCATTCTCACTGTC |
|  |  |  |  |
| PPARA[[2](#_ENREF_2)] | **197** | GACGAATGCCAAGATCTGAAAAAG | GAAGGGCGGATTGTTGTTGGTCT |
|  |  |  |  |
| PRDM16 | **91** | GAGGACATCAACACCACGAC | TGTCACGGTCACTATCCACAT |
|  |  |  |  |
| RB1 | **136** | GTGAACATCGGATCATGGAG | TTCTGGAGAGGAAGGTTGAGA |
|  |  |  |  |
| RELA | **72** | CCAGGGAGGTTCTCTTTGAG | GAACAGCTCGTCCTACCATT |
|  |  |  |  |
| SLC29A1 | **67** | GTCCGCACTATCCTCAAAAGTAT | TGGTGATGGTGAAGACGAA |
|  |  |  |  |
| ST7[[1](#_ENREF_1)] | **89** | CCTGAGCACACATCCACCCAAG | AAACAGAAACAGGGAGGGACCG |
|  |  |  |  |
| TBX1 | **84** | CTTCCAAGTGAAGCTGTTCG | TATCGCTTGTCGTCCACAG |
|  |  |  |  |
| TNFRSF9 | **105** | TCGTGTTTCTCCGTTTCTCT | CATCTTCCTCTTGGGCTGTT |
|  |  |  |  |
| TMEM26 | **198** | CGTACCAACAGAGCAGATGAT | CCCCACAAACATAAGGAGAAG |
|  |  |  |  |
| UCP1 | **114** | GAGTTCTTCACCACAGGGAAA | TCTGACCTTGACCACCTCTGT |
|  |  |  |  |
| ZIC1 | **118** | CCAACCCCAAAAAGTCGTG | TCCTCCCAGAAGCAGATGT |
|  |  |  |  |

1. Jorgensen W, Gam C, Andersen JL, Schjerling P, Scheibye-Knudsen M, Mortensen OH, Grunnet N, Nielsen MO, Quistorff B: **Changed mitochondrial function by pre- and/or postpartum diet alterations in sheep.** *Am J Physiol Endocrinol Metab* 2009, **297:**E1349-1357.

2. Lomax MA, Sadiq F, Karamanlidis G, Karamitri A, Trayhurn P, Hazlerigg DG: **Ontogenic loss of brown adipose tissue sensitivity to beta-adrenergic stimulation in the ovine.** *Endocrinology* 2007, **148:**461-468.
