# Supplementary figures and images for: Global gene expression profiling of brown to white adipose tissue transformation in sheep reveals novel transcriptional components linked to adipose remodeling
Source: BMC Genomics. 2015 Mar 19;16(1):215. doi: 10.1186/s12864-015-1405-8 (PMC4407871; doi:10.1186/s12864-015-1405-8)

# Additional file 2: Figure S1

## A

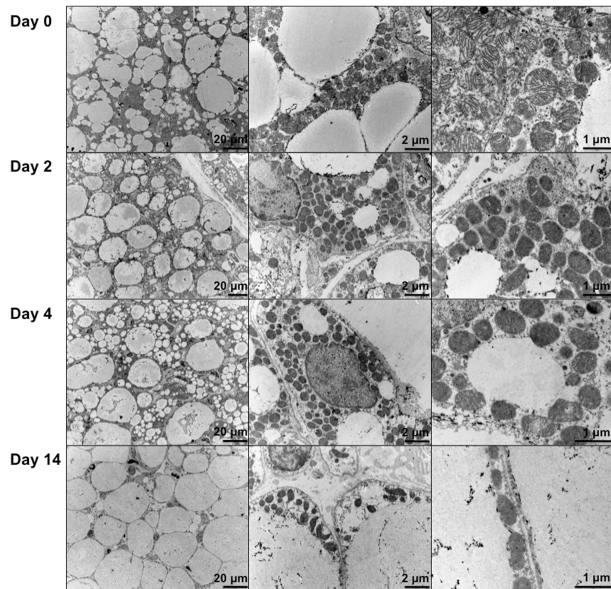

## B

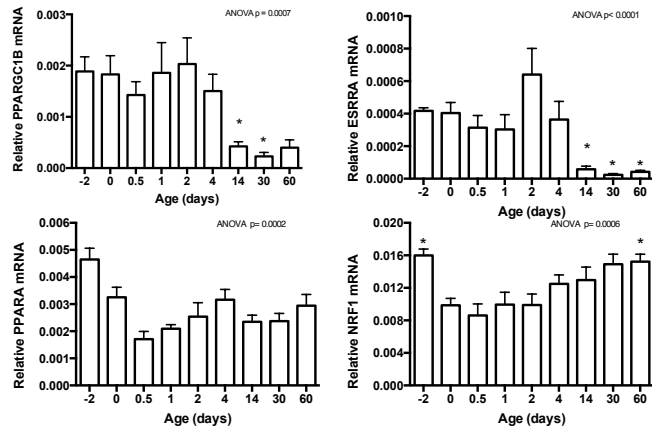

Supplement: Additional file 2: Figure S1. — Transmission electron microscopy and expression of mitochondria-related genes during brown to white adipose transformation. Transmission electron microscopy and expression of mitochondria-related genes during brown to white adipose transformation. (A) Transmission electron micrographs of perirenal adipose tissue at postnatal days 0, 2, 4 and 14. Representative images are shown for each of the indicated time points (n = 3) in three different magnifications. (B) Total RNA was isolated from perirenal adipose tissue and used for RT-qPCR analysis. Relative expression was measured for peroxisome proliferator-activated receptor γ (PPARG) co-activator 1β (PPARGC1B), PPARA, estrogen-related receptor α (ERRA) and nuclear respiratory factor 1 (NRF1). The mRNA expression levels were normalized to expression of β-actin (ACTB). Data are mean + SEM (n =4-5); *, p < 0.05 vs. day 0. [file 12864_2015_1405_MOESM2_ESM.pdf]

Additional file 7: Figure S2

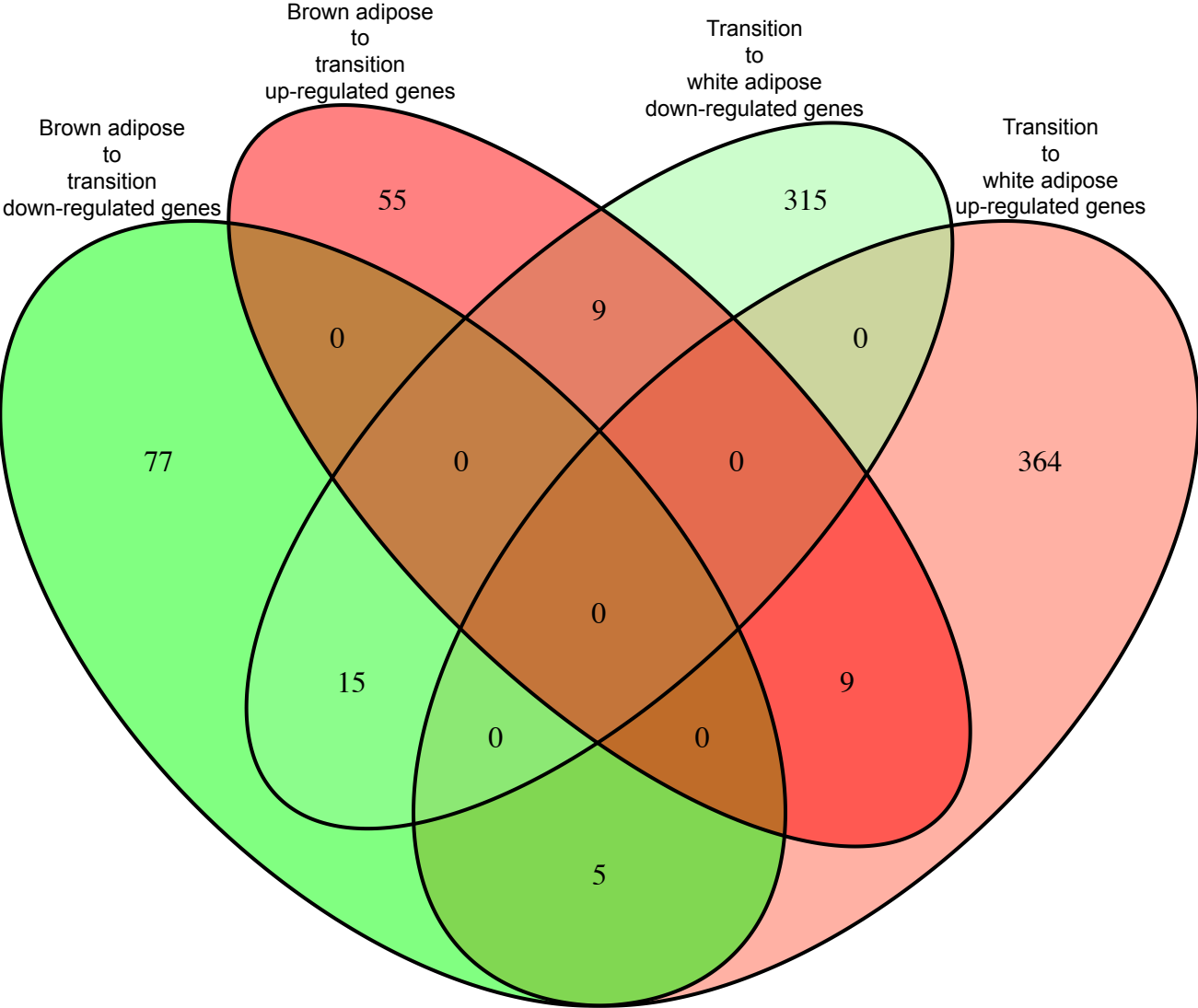

Supplement: Additional file 7: Figure S2. — Venn diagram of differentially expressed genes between the three phases. [file 12864_2015_1405_MOESM7_ESM.pdf]

Additional file 8: Figure S3

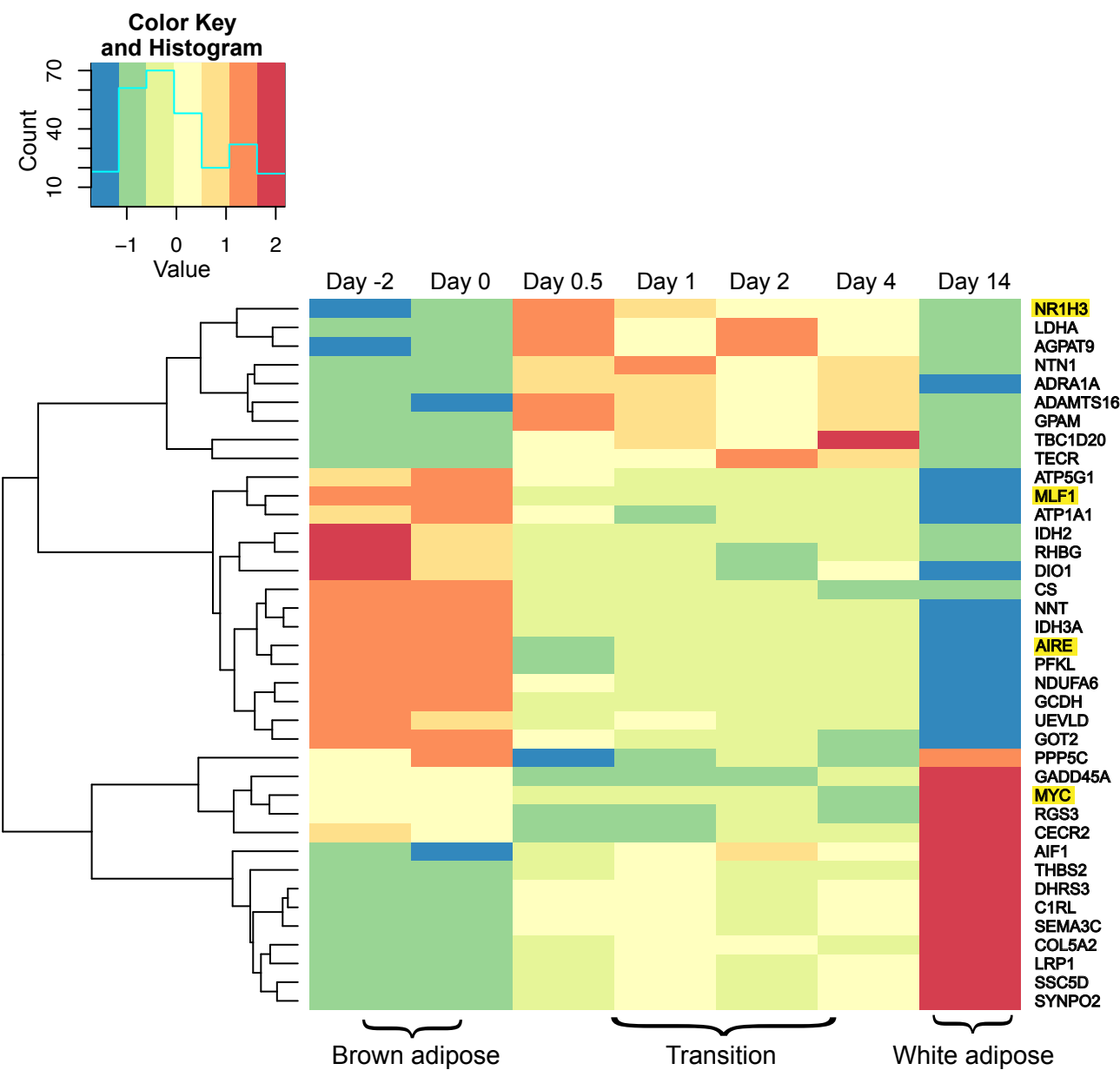

Supplement: Additional file 8: Figure S3. — Heatmap of the 38 genes regulated between the three phases. Heatmap of the 38 genes regulated between the brown adipose phase and the transition phase as well as between the transition phase and the white adipose phase. Transcription factors are highlighted in yellow. [file 12864_2015_1405_MOESM8_ESM.pdf]

# Additional file 11: Figure S4

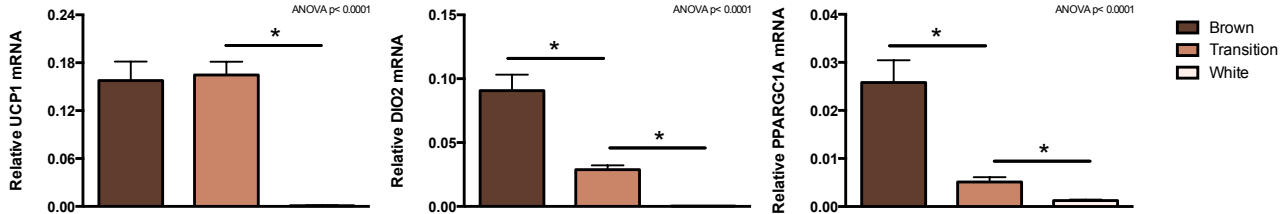

Supplement: Additional file 11: Figure S4. — Relative levels of brown adipose-associated genes during the three phases of the postnatal adipose transformation. Relative levels of brown adipose-associated genes during the three phases of the postnatal adipose transformation. These data are an alternative presentation of the data presented in Figure 1B. Total RNA was isolated from perirenal adipose tissue and used for RT-qPCR analysis. Relative expression was measured for uncoupling protein 1 (UCP1), type II iodothyronine deiodinase (DIO2) and peroxisome proliferator-activated receptor γ (PPARG) co-activator 1α (PPARGC1A). The mRNA expression levels were normalized to expression of β-actin (ACTB). Data are mean + SEM (brown, n = 9; transition, n = 20; white, n = 15); *, p < 0.05. [file 12864_2015_1405_MOESM11_ESM.pdf]

Additional file 12: Figure S5

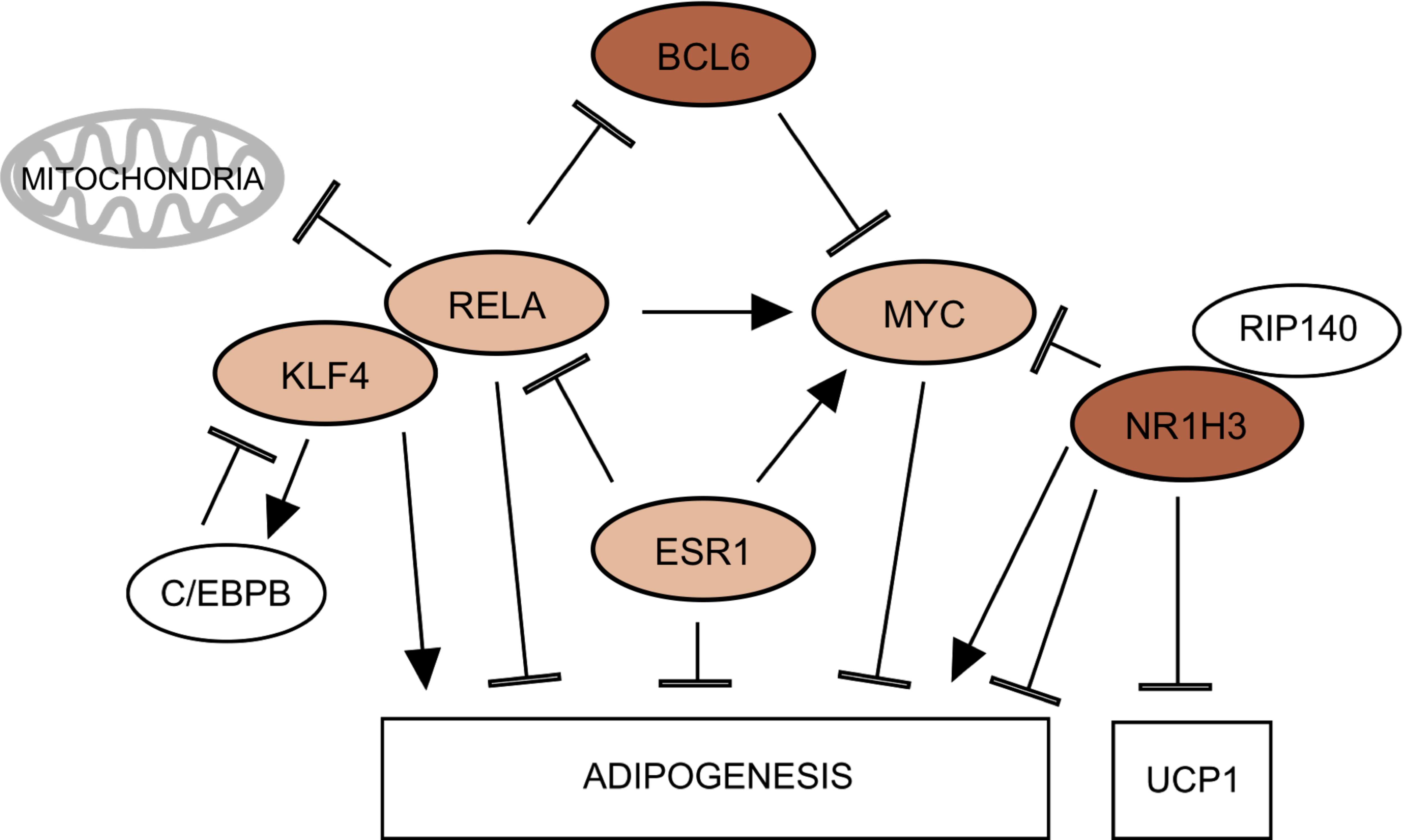

Supplement: Additional file 12: Figure S5. — Mutual interactions between differentially expressed transcription factors with putative response elements in an enriched set of regulated genes. Mutual interactions between the six differentially expressed transcription factors with putative response elements in an enriched set of genes with altered expression during the adipose transformation. The brown oval-shaped circles represent the differentially expressed transcription factors; white oval-shaped circles represent other transcriptional components functionally linked to the differentially expressed transcription factors; stop arrows indicate repression/inhibition; arrows indicate activation/stimulation. [file 12864_2015_1405_MOESM12_ESM.pdf]
