# Supplementary material for: Global gene expression profiling of brown to white adipose tissue transformation in sheep reveals novel transcriptional components linked to adipose remodeling
Source: BMC Genomics. 2015 Mar 19;16(1):215. doi: 10.1186/s12864-015-1405-8 (PMC4407871; doi:10.1186/s12864-015-1405-8)
Supplement: Additional file 3: Table S2. — Read statistics for the sequencing data when mapped and annotated to the sheep genome. A table listing the counts of raw reads from the 7 time points, reads mapped to the sheep genome, uniquely mapped reads and reads mapped to 13,963 annotated genes in the sheep genome. The table also lists the percentage of total reads in the above mentioned classes. [file 12864_2015_1405_MOESM3_ESM.docx]

**Additional file 3: Table S2.** Read statistics for the sequencing data when mapped and annotated to the sheep genome.

| Time Points | Day -2 | Day 0 | Day 0.5 | Day 1 | Day 2 | Day 4 | Day 14 |
| --- | --- | --- | --- | --- | --- | --- | --- |
| Total reads | 7232338 | 7412991 | 6597192 | 7087215 | 8148689 | 7785789 | 7694790 |
| Mapped reads | 6844509 | 6986740 | 6213617 | 6649100 | 7800652 | 7467624 | 7321378 |
| Mapped reads  (% of total reads) | 94.64 | 94.25 | 94.19 | 93.82 | 95.73 | 95.91 | 95.15 |
| Uniquely mapped reads | 3694348 | 3805071 | 3429656 | 3604692 | 4296293 | 4299881 | 4615401 |
| Uniquely mapped  (% of total reads) | 51.08 | 51.33 | 51.99 | 50.86 | 52.72 | 55.23 | 59.98 |
| Uniquely mapped  (% of mapped reads) | 53.98 | 54.46 | 55.2 | 54.21 | 55.08 | 57.58 | 63.04 |
| Reads mapped to genes | 1475752 | 1537291 | 1398727 | 1446306 | 1567557 | 1525403 | 1623127 |
| Mapped to genes  (% of total reads) | 20.4 | 20.74 | 21.2 | 20.41 | 19.24 | 19.59 | 21.09 |
