# Supplementary material for: Global gene expression profiling of brown to white adipose tissue transformation in sheep reveals novel transcriptional components linked to adipose remodeling
Source: BMC Genomics. 2015 Mar 19;16(1):215. doi: 10.1186/s12864-015-1405-8 (PMC4407871; doi:10.1186/s12864-015-1405-8)
Supplement: Additional file 9: Table S6. — Transcriptional regulators with changed expression levels between the three phases. Transcription factors and co-regulators with changed expression levels in the brown adipose to transition phase shift or in the transition to white adipose phase shift. [file 12864_2015_1405_MOESM9_ESM.docx]

**Additional file 9: Table S6.** Transcriptional regulators with changed expression levels between the three phases.

| Transcription factors and co-regulators regulated between the brown adipose phase and the transition phase | | Transcription factors and co-regulators regulated between the transition phase and the white adipose phase | | Shared transcription factors |
| --- | --- | --- | --- | --- |
| **Up-regulated** | **Down-regulated** | **Up-regulated** | **Down-regulated** |  |
| ARNTL2 | AIRE | ADNP | AIRE | AIRE |
| COPRS | BHLHE40 | ARHGAP22 | ANHX | MLF1 |
| DDX41 | CLOCK | CHD6 | BCL6 | MYC |
| DMRT2 | MLF1 | CIDEC | CHCHD3 | NR1H3 (LXRA) |
| MAFB | MYC | CIRH1A | CIDEA |  |
| NR1H3 | NCOA1 | DBP | DDX5 |  |
| TXN | PIM1 | DIDO1 | EBF2 |  |
|  | SMARCC2 (BAF170) | EPC2 | GTF2H5 |  |
|  | SMARCD3 (BAF60C) | GPBP1 | HDAC3 |  |
|  | ZNF24 | H2AFY | HSPA8 |  |
|  |  | HIF3A | KDM1A (LSD1) |  |
|  |  | KDM2A | LRPPRC |  |
|  |  | KLF4 | MED21 |  |
|  |  | MAPK3 (ERK1) | MLF1 |  |
|  |  | MTA3 | NR1C2 (PPARD) |  |
|  |  | MTDH (AEG-1) | NR1H3 (LXRA) |  |
|  |  | MYC | NR2F6 (EAR2) |  |
|  |  | NDP | RUVBL1 |  |
|  |  | NFATC2 | TBX10 |  |
|  |  | NR3A1 (ESR1) | TCEB1 |  |
|  |  | NRIP1 (RIP140) | YBX1 |  |
|  |  | PATZ1 | ZNF276 |  |
|  |  | PGE3 | ZNF414 |  |
|  |  | PRRX1 | ZNF513 |  |
|  |  | RALGPS2 | ZNF526 |  |
|  |  | RB1 | ZNF672 (ZFP672) |  |
|  |  | RBPMS |  |  |
|  |  | RELA |  |  |
|  |  | RNF25 |  |  |
|  |  | RRN3 |  |  |
|  |  | RTF1 |  |  |
|  |  | SATB2 |  |  |
|  |  | SETD3 |  |  |
|  |  | SIN3A |  |  |
|  |  | SMAD1 |  |  |
|  |  | SOX4 |  |  |
|  |  | TAF15 |  |  |
|  |  | TAF1B |  |  |
|  |  | TCF21 |  |  |
|  |  | TEX10 |  |  |
|  |  | TLE1 |  |  |
|  |  | TTF1 |  |  |
|  |  | ZNF184 |  |  |
|  |  | ZNF23 |  |  |
|  |  | ZNF268 |  |  |
|  |  | ZNF3 |  |  |
|  |  | ZNF438 |  |  |
|  |  | ZNF598 |  |  |
